# Targeting HIF-1α Regulatory Pathways as a Strategy to Hamper Tumor-Microenvironment Interactions in CLL

**DOI:** 10.3390/cancers13122883

**Published:** 2021-06-09

**Authors:** Candida Vitale, Valentina Griggio, Chiara Riganti, Maria Todaro, Joanna Kopecka, Rebecca Jones, Chiara Salvetti, Elia Boccellato, Francesca Perutelli, Claudia Voena, Laura Godio, Mario Boccadoro, Marta Coscia

**Affiliations:** 1 University Division of Hematology, A.O.U. Città della Salute e della Scienza di Torino, via Genova 3, 10126 Torino, Italy; candida.vitale@unito.it (C.V.); valentina.griggio@unito.it (V.G.); maria.todaro@unito.it (M.T.); rebecca.jones@unito.it (R.J.); chiara.salvetti@unito.it (C.S.); elia.boccellato@unito.it (E.B.); francesca.perutelli@unito.it (F.P.); mario.boccadoro@unito.it (M.B.); 2 Department of Molecular Biotechnology and Health Sciences, University of Torino, via Nizza 52, 10126 Torino, Italy; claudia.voena@unito.it; 3 Department of Oncology, University of Torino, via Santena 5, 10126 Torino, Italy; chiara.riganti@unito.it (C.R.); joanna.kopecka@unito.it (J.K.); 4 Division of Pathology, A.O.U. Città della Salute e della Scienza di Torino, via Santena 5, 10126 Torino, Italy; lgodio@cittadellasalute.to.it

**Keywords:** chronic lymphocytic leukemia, tumor microenvironment, hypoxia inducible factor-1α, CXCL12/CXCR4 axis, drug resistance

## Abstract

**Simple Summary:**

In chronic lymphocytic leukemia (CLL), the interplay between the neoplastic clone and the tumor microenvironment largely contributes to leukemia survival, tumor propagation and drug resistance. A better understanding of the molecular circuits sustaining the biological effects of this microenvironment-induced support is fundamental for designing targeted treatment strategies that can be beneficial, especially for high-risk patients who fail standard therapy. In our study, we show that the targeting of the transcription factor HIF-1α or its regulatory pathways disrupts the mutual interactions occurring between the tumor microenvironment and CLL cells and exerts anti-tumor effects, by acting both at the leukemic cell- and stromal cell-level. HIF-1α and its regulatory pathways possibly represent appealing targets in the quest for novel strategies to overcome microenvironment-mediated tumor support in CLL.

**Abstract:**

The hypoxia-inducible factor 1 (HIF-1) and the CXCL12/CXCR4 axis regulate the interaction of chronic lymphocytic leukemia cells and the tumor microenvironment. However, the interconnections occurring between HIF-1 and the CXCL12/CXCR4 axis are not fully elucidated. Here, we demonstrate that the CXCL12/CXCR4 axis plays a pivotal role in the positive regulation of the α subunit of HIF-1 (HIF-1α) that occurs in CLL cells co-cultured with stromal cells (SC). Inhibitors acting at different levels on CXCR4 downstream signalling counteract the SC-induced HIF-1α upregulation in CLL cells, also hindering the SC-mediated pro-survival effect. HIF-1α inhibition also exerts off-tumor effects on the SC component, inducing the downregulation of target genes, including *CXCL12*. Consistently, our data show that pretreatment of leukemic cells and/or SC with idelalisib effectively abrogates the SC-mediated survival support. A combined on-tumor and off-tumor inhibition of HIF-1α was also observed in idelalisib-treated patients, who showed, along with a downregulation of HIF-1α target genes in leukemic cells, a significant decrease in CXCL12 serum concentration and changes in the bone marrow microenvironment. Our data demonstrate that the targeting of HIF-1α or its regulatory pathways acts at the tumor- and SC-level, and may be an appealing strategy to overcome the microenvironment-mediated protection of CLL cells.

## 1. Introduction

The hypoxia inducible factor 1 (HIF-1) transcription factor plays a pivotal role in cellular responses to hypoxia, both in normal and in neoplastic tissues, where it is often upregulated. In tumor cells, HIF-1 supports metabolic adaptation, neoangiogenesis, cell survival and migration, with an overall disease-promoting effect [1]. HIF-1 is a heterodimer consisting of a constitutively expressed HIF-1β subunit and an inducible HIF-1α subunit [2]. HIF-1α expression and activity are regulated upon cellular oxygen concentration, but also through non-canonical regulation via the activation of multiple intracellular signalling pathways [3,4].

In chronic lymphocytic leukemia (CLL) cells, HIF-1α is constitutively expressed compared to normal B cells [5], and it acts as an important regulator of the interplay between the neoplastic clone and the tumor microenvironment. The interaction with stromal cells (SC) induces HIF-1α in CLL cells, through an increased activity of the RAS/ERK1-2, RHOA/RHOA kinase and PI3K/AKT pathways, contributing to drug resistance mechanisms, leukemia survival and tumor propagation [6,7,8]. The already reported anti-tumor effects of HIF-1α inhibition are in part the result of a perturbation of the molecular circuits sustaining microenvironment-mediated protection from apoptosis [7,8]. However, the underlying mechanisms have not been completely elucidated, and it is currently unknown whether SC-induced HIF-1α expression in CLL cells relies on direct cell-to-cell contact or soluble factors.

In CLL, the CXCL12/CXCR4 axis is one of the main players in the microenvironment-induced pro-survival support [9], and its inhibition is able to render CLL cells more susceptible to spontaneous and drug-induced apoptosis [10]. CLL cells constitutively express functional CXCR4 [11] which, upon the binding with CXCL12 (also known as stromal cell–derived factor-1, SDF-1), activates a multitude of intracellular pathways, including ERK1-2 and PI3K/AKT, whose inhibition overcomes SC-mediated drug resistance [12,13]. In the neoplastic niche, the non-leukemic cell component is also exposed to hypoxia and HIF-1α-dependent signalling, which contribute to the creation of an immunosuppressive and pro-tumor microenvironment [14]. The function of virtually all immune cell types can be directly or indirectly modulated by hypoxia [15]. Moreover, in endothelial cells, HIF-1α is crucial in driving neo-angiogenesis, overall supporting tumor growth and progression [16]. In human bone marrow (BM)-derived SC, hypoxia and subsequent HIF-1α activation have an impact on metabolic signature, differentiation, migration and proliferation [17]. However, whether HIF-1α promotes CXCL12 production by BM-derived SC of CLL patients, thus leading to CXCR4 activation and SC-induced drug-resistance in leukemic cells, is currently unknown.

The purpose of the present study was to evaluate the possible interconnections between the CXCL12/CXCR4 axis and the transcription factor HIF-1α in CLL, and to elucidate the role of HIF-1α regulatory pathways, both at the tumor cells- and SC-level, in the protection exerted by SC towards apoptosis in leukemic cells.

## 2. Materials and Methods

### 2.1. Patients’ Samples

A total of 105 patients with CLL were included in the study (Table S1). The diagnosis of CLL was made following the International Workshop on CLL-National Cancer Institute guidelines [18]. Peripheral blood (PB) or BM samples were collected after patients’ informed consent, in accordance with the Declaration of Helsinki and approval by the local Ethics Committee. Samples were collected when patients were treatment-naïve or off therapy for at least one year. In selected experiments, samples were collected from patients receiving treatment with idelalisib plus rituximab according to the approved indication: PB was collected before treatment start and after 1 and 6 months of treatment, and BM biopsies were performed for clinical purposes before treatment start and after at least 12 months of therapy.

### 2.2. Cell Lines and Cell Culture

Peripheral blood mononuclear cells (PBMC) were isolated by density gradient using Ficoll-Hypaque (Sigma-Aldrich, Milan, Italy). The anti-CD19 PerCp Vio700 and the anti-CD5-APC monoclonal antibodies (Miltenyi Biotec, Bologna, Italy) were used to evaluate the percentage of CLL cells in PBMC by flow cytometry. A FACSCalibur (Becton Dickinson, Mountain View, CA, USA) and a BD Accuri C6 flow cytometer (BD Bioscences, San José, CA, USA) were used for data acquisition. Data analysis was performed by FlowJo software (Tree Star, Inc, Ashland, OR, USA). The magnetic activated cell sorting (MACS) method (Miltenyi Biotec, Bologna, Italy) was used to purify tumor cells, when the percentage of CD19+/CD5+ cells was lower that 90%.

BM aspirate samples were lysed using a red blood cells lysis solution (Miltenyi Biotec, Bologna, Italy). Patient-derived bone marrow stromal cells (BMSC) were generated from 16 patients with CLL, as previously described [19]. The M2-10B4 murine SC line (ATCC #CRL-1972) was also used.

Serum was obtained from the centrifugation of PB samples and stored at −80 °C until use.Patient-derived cells and SC line were cultured in RPMI-1640 medium (Life Technology, Carlsbad, CA, USA) with 10% fetal bovine serum and penicillin/streptomycin (Life Technology, Carlsbad, CA, USA), at 37 °C, 5% CO_2_. CLL cells (10^6^ cells) were cultured in the presence or absence of M2-10B4 SC (5 × 10^5^ cells) and exposed to CXCL12 100 ng/mL (Human SDF-1α/CXCL12; Miltenyi Biotec, Bologna, Italy), CXCR4 antagonist AMD3100 5 µg/mL (Genzyme, Europe B.V., Naarden, The Netherlands), simvastatin 1 μM (Sigma Aldrich, Milan, Italy), ERK1-2 kinase inhibitor PD98059 1 μM (Sigma Aldrich, Milan, Italy), PI3K inhibitor idelalisib 10 μM, unless otherwise specified (Selleckchem, Munich, Germany), HIF-1α inhibitor BAY87-2243 1 μM (Selleckchem, Munich, Germany) and fludarabine 10 µM (arabinosyl-2-fluoroadenine, dephosphorylated nucleoside form of fludarabine; Sigma Aldrich, Milan, Italy) for 30 min, 6 h or 48 h. The medium of M2-10B4 SC cultured for 2 days was collected and used in CLL cells-SC 30 min co-culture. For inhibitors titration experiments, we exposed M2-10B4 SC (5 × 10^5^/mL) for 48 h to idelalisib and BAY87-2243 at indicated increasing concentrations. For each compound, the concentrations used for in vitro cell culture were selected based on our previous experience and on data from the literature [6,8,10,20,21].

The MEBCYTO-Apoptosis Kit (MBL Medical and Biological Laboratories, Nagoya, Japan) was used to assess cell viability by staining with Annexin-V/Propidium Iodide (Ann-V/PI) and flow cytometry.

### 2.3. Western Blot (WB) Analysis

The Nuclear Extract Kit (Active Motif, La Hulpe, Belgium) was used to extract cell proteins and to separate cytosolic and nuclear fractions, which were then resolved by SDS-PAGE and transferred to nitrocellulose membranes (Bio-Rad, Hercules, CA, USA). The following monoclonal antibodies were used: anti-RAS (Millipore, Bedford, MA, USA); anti-p(Thr202/Tyr204, Thr185/Tyr187)-ERK1–2 (Millipore, Bedford, MA, USA); anti-ERK1-2 (Millipore, Bedford, MA, USA); anti-p(Ser 473)AKT (Millipore, Bedford, MA, USA); anti-AKT (Millipore, Bedford, MA, USA); anti-HIF-1α (BD Biosciences, San José, CA, USA). To control the equal protein loading we used the following antibodies: anti-ACTIN (Sigma Aldrich, Milan, Italy), anti-GAPDH, anti-TUBULIN, anti-TATA-box binding protein (TBP) and anti-PCNA (all antibodies are from Santa Cruz Biotechnology Inc., Heidelberg, Germany). Secondary peroxidase-conjugated antibodies (Bio-Rad, Hercules, CA, USA) were used. The detection of the isoprenylated membrane-associated RAS protein (RAS-GTP) and total cytosolic form was per-formed by pull down-assay using the Ras Activation Assay kit (Millipore, Bedford, MA, USA) as previously described [22] and immunoblot.

Blot images were acquired with a ChemiDocTM Touch Imaging System device (Bio-Rad Laboratories, Milan, Italy). The ImageJ software (NIH, Bethesda, MD, USA) was used to perform densitometric analysis of western blot band intensity. The band intensity of the proteins of interest was normalized on the correspondent housekeeping proteins.

### 2.4. Akt and HIF-1α Activity

Akt activity was measured with the AKT Kinase Activity Assay Kit (Abcam, Cambridge, UK), as per manufacturer’s instructions. Nuclear proteins were extracted using the Nuclear Extract Kit (Active Motif, Rixensart, Belgium), and quantified. The activity of HIF-1α was assessed in nuclear extracts using the TransAM™ HIF-1α Transcription Factor Assay Kit (Active Motif, Rixensart, Belgium), according to manufacturer’s instructions. Data are expressed as U absorbance/mg cell proteins (U/mg prot).

### 2.5. RNA Extraction and Quantitative Real-Time PCR (qRT-PCR)

RNA was extracted and reverse-transcribed using the QuantiTect Reverse Transcription Kit (Qiagen, Hilden, Germany). RT-PCR was performed using IQ−SYBR Green Supermix (Bio-Rad, Hercules, CA, USA), according to the manufacturer’s instructions. The primer sequences were designed with the qPrimerDepot software (accessed date: 7 September 2020, http://primerdepot.nci.nih.gov/). The primer sequences were: *CA9*: 5′-GTGCCTATGAGCAGTTGCTGTC-3′ and 3′-AAGTAGCGGCTGAAGTCAGAGG-5′; *CXCR4*: 5′-CTCCTCTTTGTCATCACGCTTCC-3′ and 3′-GGATGAGGACACTGCTGTAGAG-5′; *CXCL12*: 5′-TGAGAGCTCGCTTTGAGTGA-3′ and 3′-CACCAGGACCTTCTGTGGAT-5′; *ENO1*: 5′-GCTCCGGGACAATGATAAGA-3′, 5′-TCCATCCATCTCGATCATCA-3′; *GLUT1*: 5′-CCTGCAGTTTGGCTACAACA-3′ and 3′-TAACGAAAAGGCCCACAGAG-5′; *S14* (housekeeping): 5′-GGTGCAAGGAGCTGGGTAT-3′ and 3′-TCCAGGGGTCTTGGTCCTATTT-5′; *VEGF*: 5′-ATCTTCAAGCCATCCTGTGTGC-3′, 5′-GCTCACCGCCTCGGCTTGT-3′. The comparative CT method was used to calculate *CA9*, *CXCR4*, *CXCL12*, *GLUT1*, *ENO1* and *VEGF* expression relative to *S14* product, used as a housekeeping gene, with the Bio-Rad Software Gene Expression Quantitation (Bio-Rad, Hercules, CA, USA).

### 2.6. CXCL12 Quantification

CXCL12 was measured on patients’ serum using the human CXCL12/SDF1α Quantikine ELISA kit (R&D Systems, Minneapolis, MN, USA), according to the manufacturer’s instructions. For each experiment, a titration curve was prepared using serial dilutions of the standard CXCL12 of the kit. The curve was then used to extrapolate the CXCL12 concentration in the samples.

### 2.7. Immunohistochemistry

Three-µm-thick sections from Bouin’s solution-fixed, paraffin-embedded BM biopsies were stained with haematoxylin-eosin and immunostained with an automated stainer device (Ventana-Ultra, Ventana Medical Systems, Tucson, AZ, USA) using polyclonal antibodies against CD34 (clone QBEnd/10; #NCL-L-END; Novocastra; Leica Microsystems, Milton Keynes, UK) at a 1:50 dilution at 37 °C for 36 min, and CD68 (clone PG-M1, cod. M0876; Dako, Carpinteria, CA, USA) at a 1:50 dilution at 25 °C for 30 min. Sinusoid-like vessel density and CD68+ cellular extensions were analyzed in 10 random high power fields employing 40× magnification using a standard light microscope (Leica, Wetzlar, Germany).

### 2.8. Statistical Analysis

GraphPad Prism software (version 6.01, San Diego, CA, USA) was used to perform statistical analysis of data (paired and unpaired *t*-test). Results are expressed as mean ± standard error of the mean (SEM), unless otherwise specified. Statistical significance was defined as a *p*-value <0.05.

## 3. Results

### 3.1. CXCL12/CXCR4 Axis Is a Main Regulator of SC-Induced HIF-1α Activation in CLL Cells

**Figure 1:**
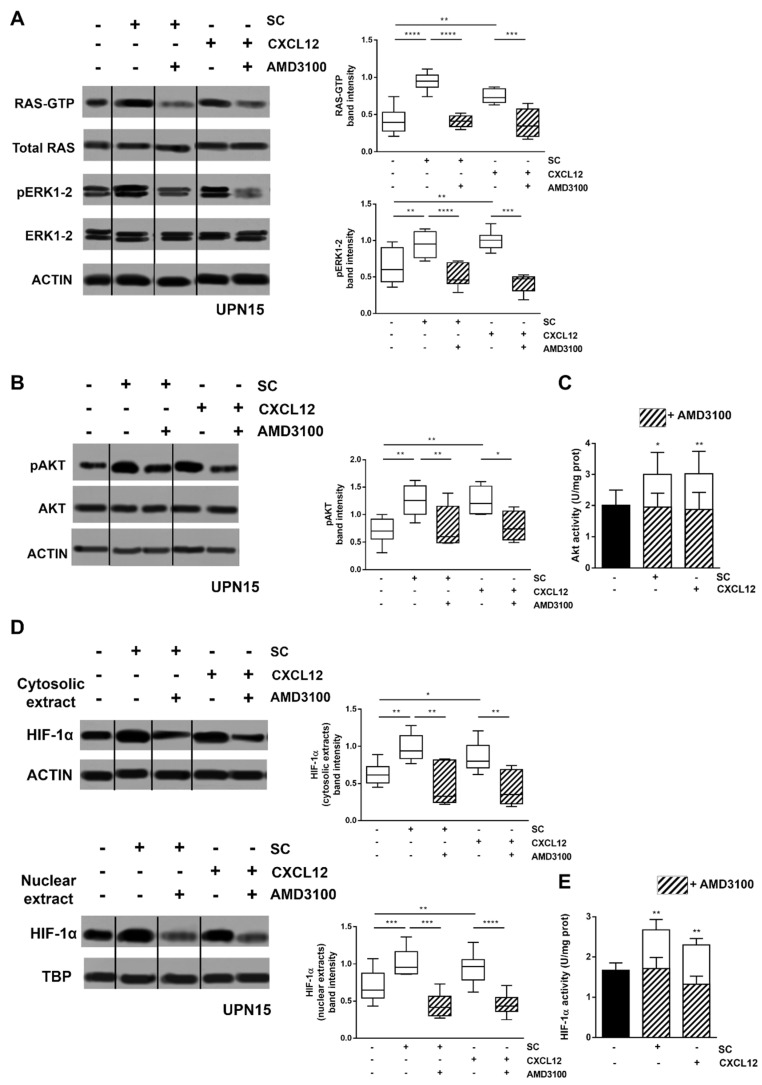
The CXCL12/CXCR4 axis plays a central role in the SC-mediated triggering of HIF-1α regulatory pathways. Primary CLL cells were cultured for 48 h in presence of M2-10B4 SC or CXCL12. In selected conditions, the CXCR4 antagonist AMD3100 was added. Both SC and CXCL12 induced an increase in the amount of GTP-bound RAS (RAS-GTP) and of the active phosphorylated form of ERK1-2 (pERK1-2) (**A**), and in the phosphorylation and activity of AKT (**B**,**C**). Accordingly, CLL cells cultured with SC or CXCL12 displayed an increase in the cytosolic and nuclear amount of HIF-1α (**D**), and in HIF-1α activity (**E**). The addition of the CXCR4 antagonist AMD3100 abrogated the inducing effects mediated both by SC and CXCL12 at all levels. In (**A**,**B**,**D**) a representative blot (with relative Unique Patient Number, UPN), together with the corresponding cumulative band intensity data of 6 independent experiments, respectively, is shown. Box and whiskers plots show median values, 25–75% percentiles, and minimum and maximum values for each group. In (**C**,**E**) bar graphs represent mean results and SEM (*n* = 6). Repositioned gel lanes are indicated by vertical lines. **** *p* < 0.0001, *** *p* < 0.001, ** *p* < 0.01 and * *p* < 0.05. Please find the whole western blot in the Supplementary File 1.

We have previously demonstrated that exposure of CLL cells to SC induces the activation of RAS/ERK1-2 and PI3K/AKT signalling pathways, leading to an upregulation of the downstream transcription factor HIF-1α [6]. Here, we show that this SC-induced upregulation is mainly mediated by the CXCL12/CXCR4 axis. Indeed, after 48 h culture, the CXCR4 antagonist AMD3100 was capable of abrogating the SC-induced activation of RAS/ERK1-2 and PI3K/AKT signalling, and the downstream increase in HIF-1α amount and transcriptional activity (*p* < 0.05) (Figure 1). This finding was further corroborated by the observation that exposure to CXCL12 substantially recapitulated the stimulation exerted by SC on RAS/ERK1-2 and PI3K/AKT signalling, and on HIF-1α accumulation and activity. Again, these effects were significantly and almost fully counteracted by the CXCR4 antagonist AMD3100 (*p* < 0.05) (Figure 1). The ability of AMD3100 to block the CXCL12/CXCR4 axis was already evident after 6 h exposure, and to a lesser extent after 30 min, confirming that HIF-1α inhibition is a targeted effect rather than being a consequence of cell death potentially induced by AMD3100 (Figures S1–S3).

### 3.2. Inhibitors of CXCR4 Downstream Signalling Effectively Counteract SC- and CXCL12-Induced HIF-1α Upregulation in CLL Cells

**Figure 2:**
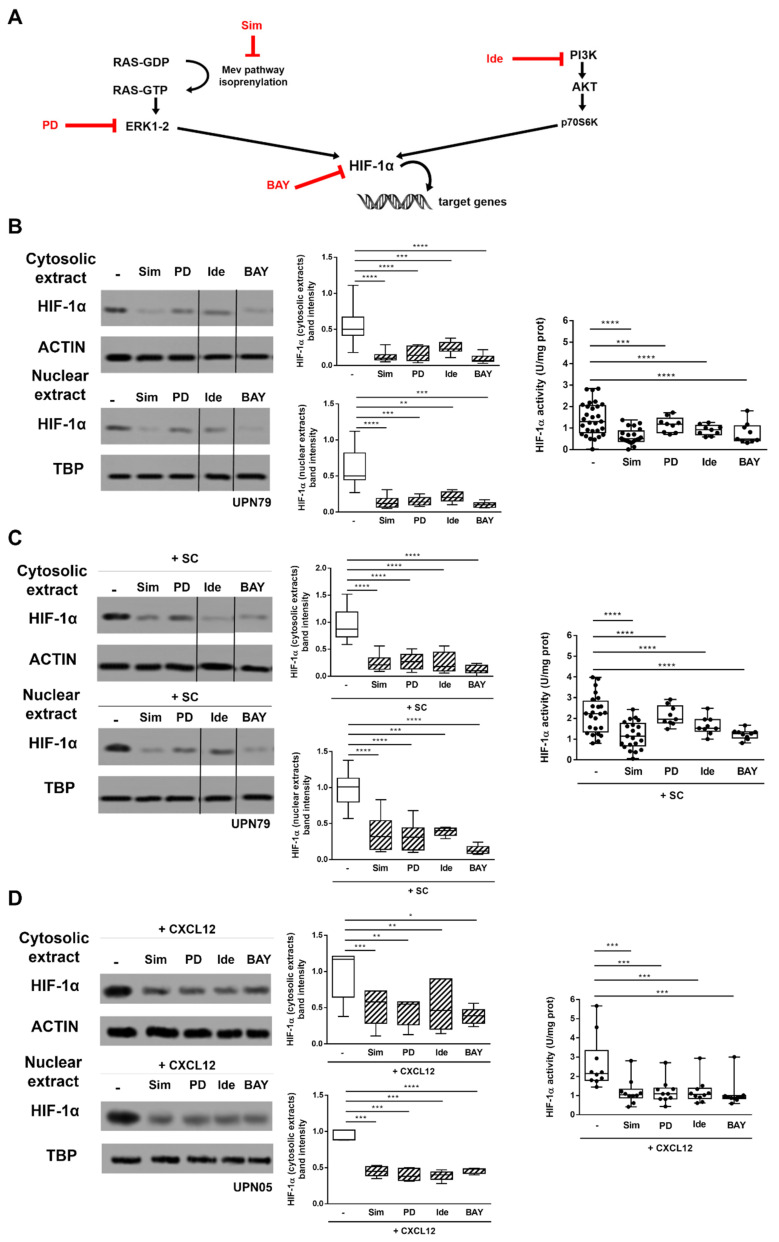
Targeting the RAS/ERK1-2 and PI3K/AKT signalling inhibits SC- and CXCL12-induced HIF-1α upregulation. A schematic representation of RAS/ERK1-2 and PI3K/AKT HIF-1α regulatory pathways, and the target protein of each inhibitor used in the following experimental setting (i.e., simvastatin, Sim; PD98059, PD; idelalisib, Ide; BAY87-2243, BAY) is depicted in (**A**). CLL cells cultured for 48 h in the absence (**B**) and in presence of M2-10B4 SC (**C**) or CXCL12 (**D**) were exposed to simvastatin, PD98059, idelalisib or BAY87-2243 and evaluated for HIF-1α expression and activity. Treatment with targeted inhibitors reduced the cytosolic and nuclear amount of HIF-1α and downregulated its activity, both in the absence and in the presence of SC or CXCL12. In (**B**–**D**) a representative blot with UPN and cumulative band intensity data obtained from the analysis of 9, 9 and 5 independent experiments, respectively, is shown. Repositioned gel lanes are indicated by vertical lines. Box and whiskers plots show median values, 25–75% percentiles, and minimum and maximum values for each group; each point represents a single sample. **** *p* < 0.0001, *** *p* < 0.001, ** *p* < 0.01 and * *p* < 0.05. Please find the whole western blot in the Supplementary File 1.

We tested whether the inhibitors of RAS (i.e., simvastatin), ERK1-2 (i.e., PD98059) and PI3K/AKT (i.e., idelalisib), acting at different levels of the signalling pathways, affected SC- and CXCL12-induced HIF-1α upregulation in primary CLL cells (Figure 2A). When freshly isolated leukemic cells were cultured with simvastatin, PD98059, idelalisib, as well as with HIF-1α inhibitor BAY87-2243, we observed a decrease in HIF-1α amount and activity (*p* < 0.001) (Figure 2B). Interestingly, the same inhibitory effect was maintained also when intracellular pathways were upregulated by co-culture with SC (*p* < 0.0001) (Figure 2C) or exposure to CXCL12 (*p* < 0.001) (Figure 2D). These data demonstrate that CXCR4-dependent signalling pathways can be effectively targeted at different levels thus hindering the CXCL12-induced accumulation and activation of HIF-1α.

### 3.3. The Targeted Inhibition of HIF-1α Regulatory Pathways Hinders the SC-Mediated Protection from Spontaneous and Fludarabine-Induced Cell Death

**Figure 3:**
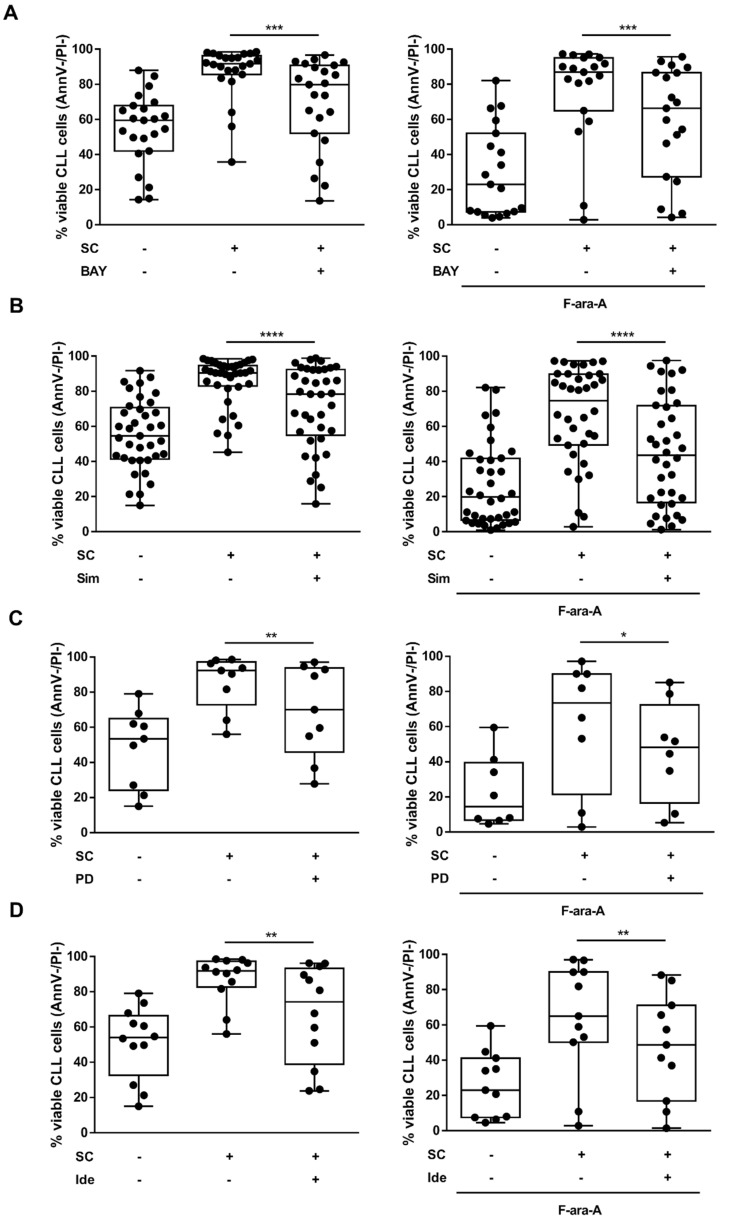
Inhibitors of HIF-1α regulatory pathways counteract SC protection toward spontaneous and fludarabine-induced leukemic cell death. CLL cells were cultured alone or in the presence of M2-10B4 SC. In all experiments, the co-culture with SC resulted in a significantly higher viability as compared to CLL cells cultured alone. Cell cultures were exposed to BAY87-2243 (BAY), simvastatin (Sim), PD98059 (PD) or idelalisib (Ide), alone or in association with fludarabine (F-ara-A). Cell viability was evaluated after 48 h treatment. The addition of BAY87-2243 (**A**), simvastatin (**B**), PD98059 (**C**) and idelalisib (**D**) to CLL/SC co-cultures significantly reduced the viability of CLL cells compared to untreated co-cultures, both in the absence and in the presence of fludarabine (left and right panels, respectively). Box and whiskers plots show median values of alive CLL cells percentages, considered as Ann-V-negative and PI-negative (AnnV-/PI-), 25–75% percentiles, and minimum and maximum values for each group; each point represents a single sample value (*n* = 23 and *n* = 19 in (**A**); *n* = 37 and *n* = 36 in (**B**); *n* = 9 and *n* = 8 in (**C**); *n* = 12 and *n* = 11 in (**D**)). **** *p* < 0.0001, *** *p* < 0.001, ** *p* < 0.01 and * *p* < 0.05.

SC are known to protect CLL cells from spontaneous apoptosis and fludarabine-induced cytotoxicity. HIF-1α expression correlates with in vitro resistance to fludarabine and the HIF-1α inhibitor BAY87-2243 enhances fludarabine cytotoxicity [8]. Therefore, we wondered whether the inhibition of HIF-1α regulatory pathways may exert similar effects. In line with previous data, we observed that BAY87-2243 was effective in counteracting the SC-mediated protection toward spontaneous and fludarabine-induced cell death (*p* < 0.001) (Figure 3A). Of note, simvastatin, PD98059 and idelalisib also produced a significant decrease in leukemic cells’ viability, partially abrogating the protective effect exerted by SC toward spontaneous and fludarabine-induced cell death (*p* < 0.05) (Figure 3B–D).

### 3.4. The Inhibition of PI3K/AKT Pathway and Downstream HIF-1α Impairs CXCL12 Production in SC

**Figure 4:**
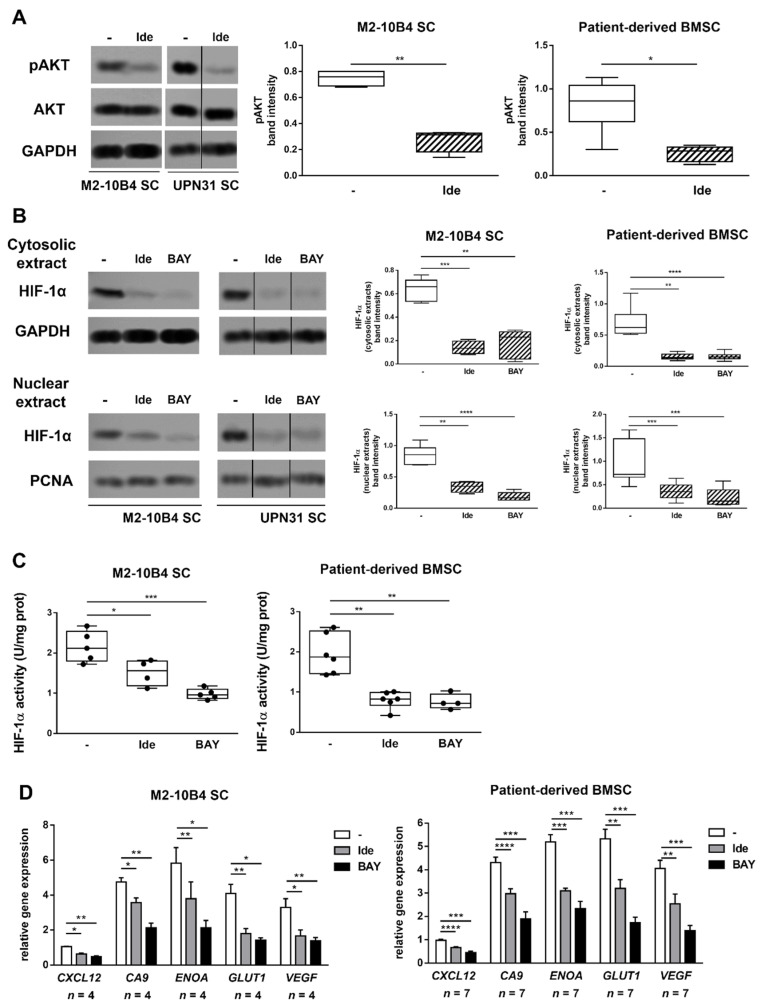
The targeting of PI3K/AKT signalling pathway downmodulates HIF-1α and its target genes expression in SC. M2-10B4 SC and patient-derived BMSC were cultured in absence or in presence of idelalisib (Ide) or BAY87-2243 (BAY) for 48 h. Idelalisib effectively inhibits pAKT expression in SC (**A**). Both drugs induced a decrease in cytosolic and nuclear HIF-1α expression (**B**), and in HIF-1α activity (**C**) after 48 h culture. *CXCL12*, *CA9*, *ENOA*, *GLUT1* and *VEGF* mRNA were also quantified, showing that idelalisib and BAY87-2243 significantly reduced HIF-1α target genes’ expression in SC (**D**). In (**A**), a representative blot (with relative UPN, when applicable) with cumulative band intensity data obtained from the analysis of 4 replicates for M2-10B4 SC and 7 patient-derived BMSC independent experiments is shown. In (**B**), a representative blot (with UPN, when applicable) with cumulative band intensity data obtained from the analysis of 6 replicates for M2-10B4 SC and 11 patient-derived BMSC independent experiments is shown. In (**A**–**C**) box and whiskers plots show median values, 25–75% percentiles and minimum and maximum values for each group. In (**C**) each point represents one experiment with M2-10B4 SC (*n* = 5) or one patient-derived SC sample (*n* = 6). In (**D**), bar graphs represent mean results obtained from the analysis of 4 replicates for M2-10B4 SC and 7 patient-derived BMSC independent experiments, together with SEM. Repositioned gel lanes are indicated by vertical lines. **** *p* < 0.0001, *** *p* < 0.001, ** *p* < 0.01 and * *p* < 0.05. Please find the whole western blot in the Supplementary File 1.

PI3Kδ is expressed and functional in SC from patients with CLL, where it plays a role in regulating CLL-SC interactions [23]. Therefore, we next investigated whether the targeting of PI3K/AKT signalling pathway could modulate HIF-1α, not only in the tumor clone but also in SC. To this aim, the SC line M2-10B4, as well as SC derived from the BM of CLL patients, were cultured alone or in the presence of idelalisib or BAY87-2243. Neither idelalisib nor BAY87-2243 affected the viability and morphology of SC after 48 h culture (data not shown). Results from our experiments showed that idelalisib is able to decrease the active form of AKT in SC, and that both idelalisib and BAY87-2243 effectively reduce the cytosolic and nuclear amount of HIF-1α, also impairing its transcriptional activity (*p* < 0.01) (Figure 4A–C). Consequently, we found that 48 h in vitro exposure of SC to idelalisib or BAY87-2243 significantly reduces the expression of the HIF-1α target genes *CXCL12*, *CA9*, *ENOA*, *GLUT1* and *VEGF* (*p* < 0.001) (Figure 4D). Titration experiments confirmed that exposure of SC to increasing concentrations of idelalisib determines a parallel progressive decrease in HIF-1α levels (Figure S4A). A dose-dependent reduction in HIF-1α expression was also evident when SC were treated with different concentrations of BAY87-2243 (Figure S4B).

### 3.5. Idelalisib Hampers Stroma-Derived Survival Signals by Targeting HIF-1α at the SC- and CLL Cell-Level

**Figure 5:**
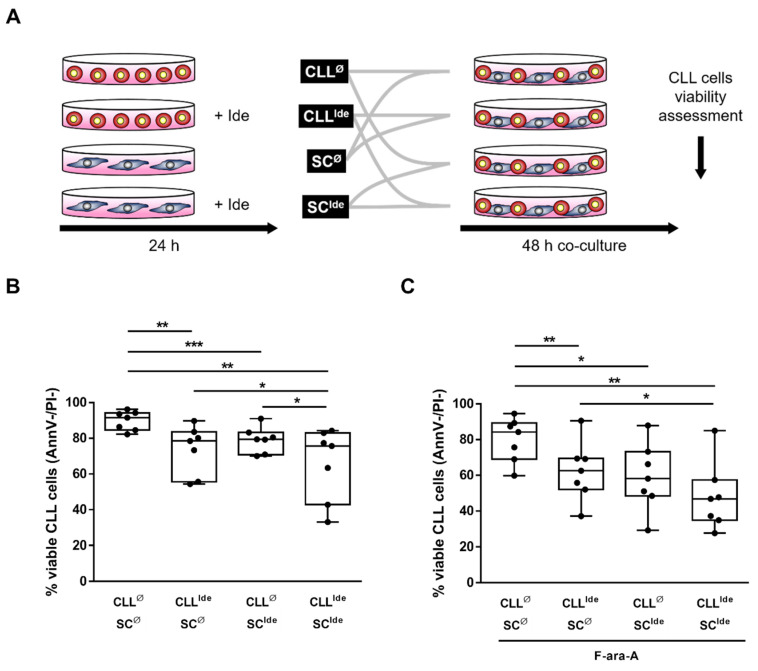
Idelalisib overcomes SC-mediated CLL cell protection bilaterally, acting at the leukemic cell- and SC-level. A schematic representation of the experiment design is depicted in (**A**). CLL cells and M2-10B4 SC were left untreated (CLL^Φ^ and SC^Φ^) or exposed to idelalisib (CLL^Ide^ and SC^Ide^) for 24 h, washed, and then co-cultured in different combinations (CLL^Φ^ + SC^Φ^, CLL^Ide^ + SC^Φ^, CLL^Φ^ + SC^Ide^, CLL^Ide^ + SC^Ide^) for an additional 48 h, with or without fludarabine (F-ara-A). The final CLL cell viability was evaluated. Pretreatment with idelalisib of CLL cells, SC or both, significantly reduced the cell viability compared to the untreated combination, both in the absence (**B**) and in the presence of fludarabine (**C**). Box and whiskers plots represent median values of alive cells percentages, considered as Ann-V-negative and PI-negative (AnnV-/PI), 25–75% percentiles and minimum and maximum values for each group; each point represents a single sample value (*n* = 7). *** *p* < 0.001, ** *p* < 0.01 and * *p* < 0.05.

We then investigated whether the ability of idelalisib to hamper the SC-mediated protection toward leukemic cell death also relies on its off-tumor effects on the stromal counterpart. To this aim, CLL cells and M2-10B4 SC were left untreated or exposed to idelalisib for 24 h, and then co-cultured for additional 48 h in the absence or presence of fludarabine (as outlined in Figure 5A). In CLL cells pre-treated with idelalisib, before co-culturing them with untreated SC, we observed a significant decrease in 48 h cell viability, both in the absence and in the presence of fludarabine (*p* < 0.01). Interestingly, pretreatment with idelalisib of the sole SC component also determined a significant increase in spontaneous and fludarabine-induced leukemic cell death after 48 h of co-culture (*p* < 0.05). Consistently, pre-treatment of both components (i.e., leukemic cells and SC) with idelalisib produced a further reduction in cell viability after 48 h of co-culture compared to the pre-treatment of each single component (*p* < 0.01) (Figure 5B,C).

### 3.6. Treatment with Idelalisib Affects HIF-1α Expression and Activity in CLL Patients

**Figure 6:**
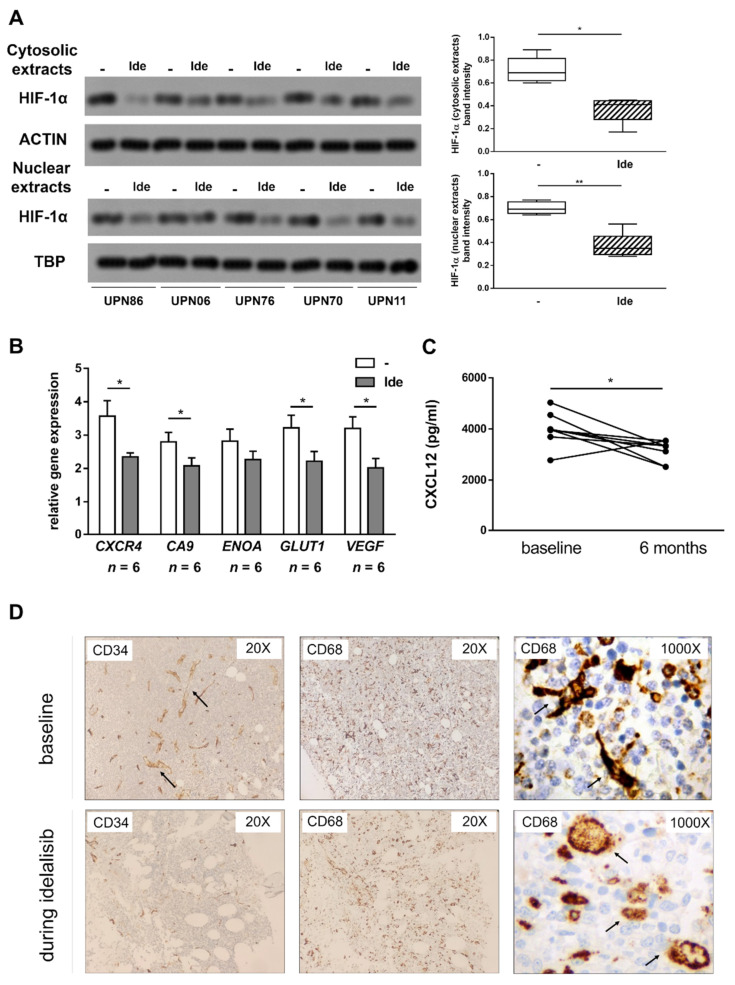
Idelalisib treatment downregulates HIF-1α in CLL cells, reduces serum CXCL12 and modifies the BM microenvironment. Leukemic cells isolated from CLL patients treated for one month with idelalisib were evaluated for HIF-1α expression by WB and for the expression of the HIF-1α target genes *CXCR4, CA9, ENOA, GLUT1* and *VEGF* by RT-PCR. Compared to the baseline, a reduced cytosolic and nuclear expression of HIF-1α (**A**), and a decreased expression of the analyzed target genes (**B**) were detected. In patients’ sera, CXCL12 concentration decreased after 6 months of treatment with idelalisib (**C**). Immunohistochemical analyses performed on BM sections collected from CLL patients during idelalisib treatment showed, compared to the baseline, a reduced vessel density, highlighted by anti-CD34 immunostaining, and an enrichment in the CD68+ monocytes/macrophages characterized by a round shape morphology and no cellular extensions (**D**). In (**A**), blots from the analysis of 5 independent experiments (with UPN) and cumulative band intensity data obtained are shown. In (**B**), bar graphs represent mean results obtained from 6 experiments together with SEM. In (**C**), a line graph represents individual data values for the same sample in each timepoint. In (**D**), immunohistochemistry of a representative experiment out of 3 is shown. ** *p* < 0.01 and * *p* < 0.05. Please find the whole western blot in the Supplementary File 1.

To corroborate our in vitro findings, we collected PBMC and serum samples from patients with CLL before and during idelalisib treatment. In all analyzed cases, the HIF-1α amount in CLL cells was consistently reduced after 1 month of treatment, as compared to the baseline (Figure 6A). According to the reduced level of HIF-1α, the expression of *CXCR4*, *CA9*, *ENOA*, *GLUT1* and *VEGF* target genes in CLL cells was impaired (Figure 6B). Notably, in line with in vitro data, we also observed a significant reduction in the serum levels of CXCL12 after 6 months of idelalisib treatment, compared to the baseline (*p* < 0.05) (Figure 6C). We also analyzed BM biopsy samples collected before and during idelalisib treatment from three CLL patients. We observed a reduction in the sinusoidal-like vessel density after idelalisib therapy compared to the baseline. In addition, the CD68+ monocyte-macrophage component, which is characterized by a round-shape morphology in the absence of cellular extensions, was enriched in the BM of idelalisib-treated patients compared to baseline samples, where dendritic cells were instead predominant (Figure 6D).

## 4. Discussion

In this study, we investigated the role of HIF-1α and its regulatory pathways in the interactions between CLL cells and their protective tumor microenvironment. We demonstrated that, in leukemic cells, the CXCL12/CXCR4 axis plays a central role in the SC-induced modulation of HIF-1α through the activation of RAS/ERK1-2 and PI3K/AKT signalling pathways. Interestingly, HIF-1α inhibition also affects the SC component, resulting in a transcriptional downregulation of several target genes, including *CXCL12*. Therefore, the targeting of HIF-1α acts both at the leukemic cell- and SC-level, abrogating the pro-survival effect exerted by stroma interactions on CLL cells and also counteracting the protection toward fludarabine-induced cell death. According to this finding, the targeting of PI3K/AKT pathway through idelalisib results in a dual effect in patients with CLL: an on-tumor effect leading to a reduced expression of HIF-1α and its target genes in leukemic cells, and an off-tumor effect leading to decreased concentrations of CXCL12 in patients’ sera.

HIF-1α is overexpressed in CLL cells [5], and this overexpression is more pronounced in cells carrying unfavorable biological characteristics such as unmutated immunoglobulin heavy chain variable region genes (IGHV) or *TP53* disruption [6,8]. In CLL, HIF-1α fosters different tumor-promoting mechanisms: it mediates the adaptation of leukemic cells to hypoxia, functions as a pro-survival factor and is implicated in drug-resistance mechanisms [6,8,24]. In addition, HIF-1α has shown to critically regulate several genes, such as *CXCR4*, involved in mediating homing and retention of CLL cells into the BM and spleen [7]. Within these tissues, leukemic cells interact with SC and other microenvironmental elements, which are known to protect them from spontaneous apoptosis and confer resistance to variety of drugs, including chemotherapy [6,8,21] and targeted drugs [25,26]. We have already reported that both hypoxia and the co-culture with SC induce in CLL cells a further increase in the nuclear expression and transcriptional activity of HIF-1α resting levels [6,8]. From the molecular standpoint, this HIF-1α upregulation is mediated by the activation of defined molecular circuits within the leukemic cells: the RAS/ERK1-2 and PI3K/AKT signalling pathways. Nonetheless, whether SC induce HIF-1α upregulation through a cell–cell contact or by paracrine factors is so far unknown.

The CXCL12/CXCR4 axis plays a leading role in the CLL cell-tumor microenvironment interactions [9]. The CXCR4 antagonist AMD3100 affects pseudoemperopolesis, migration and prosurvival signals induced by CXCL12 on CLL cells [27]. In CLL, the role of ERK1-2 and AKT as downstream signal transducers of CXCL12/CXCR4 axis has been postulated by different authors [12,28,29], but a complete overview of the CXCR4 transduction pathway is still lacking. Our data show that, in CLL cells, the SC-induced upregulation of the intracellular pathways leading to an increased expression and transcriptional activity of HIF-1α (i.e., RAS/ERK1-2 and PI3K/AKT signalling) is fully recapitulated by exposure of leukemic cells to CXCL12, and is completely abrogated by the CXCR4 antagonist AMD3100. This latter observation provides a more comprehensive characterization of the molecular circuits downstream to CXCR4 at the leukemic cell level.

It has been previously reported that *CXCR4* expression in CLL cells is under the transcriptional control of HIF-1α [7]. This observation, together with our data showing that CXCR4 signalling in turn regulates HIF-1α expression and transcriptional activity, corroborate the hypothesis of a reciprocal interaction between CXCL12/CXCR4 axis and HIF-1α in regulating the interactions between CLL cells and SC. Our findings thus support the notion of HIF-1α as a key regulator of the interactions of CLL neoplastic cells with SC. Although CXCL12 is undoubtedly a central molecule in SC-mediated HIF-1α upregulation, we cannot rule out a complementary role of cell–cell contact interactions, which should be therefore investigated to gain a full overview of the mechanisms regulating HIF-1α in CLL cells.

We next investigated whether HIF-1α also played a role in controlling the tumor supportive functions of the SC compartment. It has been shown that *CXCL12* gene expression in endothelial cells is regulated by the transcription factor HIF-1α, and that this chemokine is selectively expressed in a model of soft-tissue ischemia in vivo, in direct proportion to reduced oxygen tension [30]. Additionally, treatment of hypercholesterolemia with high-doses statins results in a decrease in circulating CXCL12 levels, which are inversely correlated with the administered dose of statin [31]. More recently, Ali et al. showed that PI3Kδ is expressed and functional in SC from both healthy donors and patients with CLL, where it plays a role in regulating CLL-stromal cell interactions [23], although they did not propose an explanatory mechanism for their observation. As far as we know, this is the first article demonstrating that CXCL12 production by SC is under the transcriptional control of HIF-1α. Indeed, the modulation of HIF-1α with its specific inhibitor BAY87-2243, or through the upstream targeting of PI3K with idelalisib, results in a transcriptional downregulation of *CXCL12*, together with several other target genes implicated in cell migration, metabolism and angiogenesis. In keeping with the hypothesis of a wide-ranging beneficial effect of antagonizing HIF-1α in the non-leukemic microenvironment, previous findings show that hypoxia-driven HIF-1α overexpression impairs the function of a variety of immune populations and causes hematopoietic dysfunctions in the BM of CLL patients [14,32].

As above mentioned, the role of SC and CXCL12/CXCR4 axis in conferring drug resistance to CLL cells has been repeatedly demonstrated [10,21]. We have recently shown that the targeting of HIF-1α with BAY87-2243 is effective in overcoming the intrinsic *TP53*-dependent and the SC-induced fludarabine resistance of CLL cells [8]. In line with these previous findings, here, we observed that the inhibition of HIF-1α regulatory pathways by simvastatin, idelalisib or PD98059 is paralleled by a reduction in CLL cells’ viability and an increased sensitivity to fludarabine. Of note, pretreatment of CLL cells and/or SC with idelalisib counteracts the protection exerted by SC toward spontaneous cell death and fludarabine-induced cytotoxicity observed in co-culture conditions. These data corroborate the notion that HIF-1α bilaterally orchestrates tumor-microenvironment interactions by controlling, on one side, the SC-mediated production of CXCL12, and on the other side, the final pro-survival activity exerted by this chemokine on leukemic target cells. In line with this hypothesis, generated on the basis of our in vitro data, HIF-1α regulatory pathways can be simultaneously targeted in the stromal and neoplastic compartment with the aim of disrupting their pro-tumor cooperation. Interestingly, this dual action on HIF-1α, which reflects on-tumor and off-tumor effects, was also confirmed on a small cohort of idelalisib-treated patients, who showed, in parallel to a decreased expression of the pro-survival factor HIF-1α in the leukemic cell compartment, a reduction in CXCL12 serum concentrations and a modification of the monocytic and endothelial architecture in the BM microenvironment. The anti-tumor effect exerted by idelalisib through HIF-1α inhibition in cancer cells, as well as in SC, would certainly need a validation in a larger cohort of CLL patients.

## 5. Conclusions

Taken together, our results confirm the central role of HIF-1α in the interactions between the tumor microenvironment and CLL cells, also elucidating the interplay with the CXCL12/CXCR4 axis. We also show that the disruption of these mutual interactions through the targeting of HIF-1α or its regulatory pathways exerts anti-tumor effects by acting at both the leukemic cell- and SC-levels, possibly representing an appealing strategy for overcoming microenvironment-mediated tumor support.

## Data Availability

The data that support the findings of this study are available from the corresponding author, M.C., upon reasonable request.

## Supplementary Materials

The following are available online at https://www.mdpi.com/article/10.3390/cancers13122883/s1. “Supplementary Information”, including Table S1: Summary of patients’ characteristics; Figure S1: The CXCL12/CXCR4 axis plays a central role in the SC-mediated triggering of HIF-1α regulatory pathways; Figure S2: The exposure of CLL cells to AMD3100 does not reduce tumor cell viability; Figure S3: The CXCL12/CXCR4 axis plays a central role in the SC-mediated triggering of HIF-1α regulatory pathways; Figure S4: Increasing concentrations of idelalisib or BAY87-2243 determine a progressive reduction of HIF-1α levels in SC. “Supplementary File 1”, including the whole blots for the representative experiments showed in the manuscript.
