# Supplementary material for: Targeting HIF-1α Regulatory Pathways as a Strategy to Hamper Tumor-Microenvironment Interactions in CLL
Source: Cancers (Basel). 2021 Jun 9;13(12):2883. doi: 10.3390/cancers13122883 (PMC8229189; doi:10.3390/cancers13122883)
Supplement: Supplementary file 1 [file cancers-13-02883-s001.zip › Supplemetal File 1_FINAL.pdf]

Western blots

Figure 1, Panel A

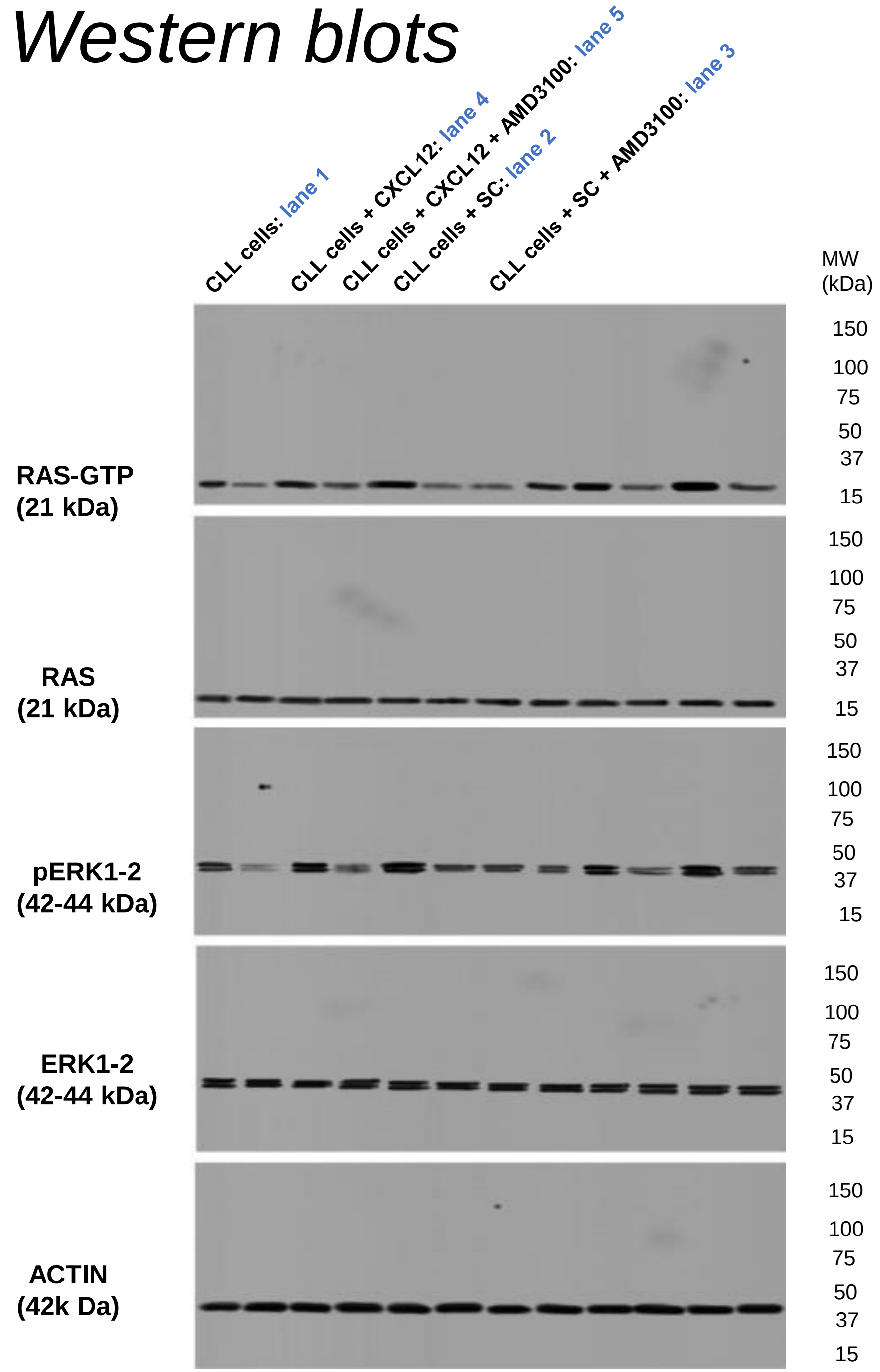

Densitometry reading

UPN15	-	SC	SC + AMD3100	CXCL12	CXCL12 + AMD3100
RAS-GTP	85,42	140,19	61,45	116,59	74,58
pERK1-2	100,89	145,92	84,76	135,19	84,76
ACTIN	115,05	126,19	117,45	134,12	134,9

The lanes of the whole blot used for each figure are indicated

Western blots

Figure 1, Panel B

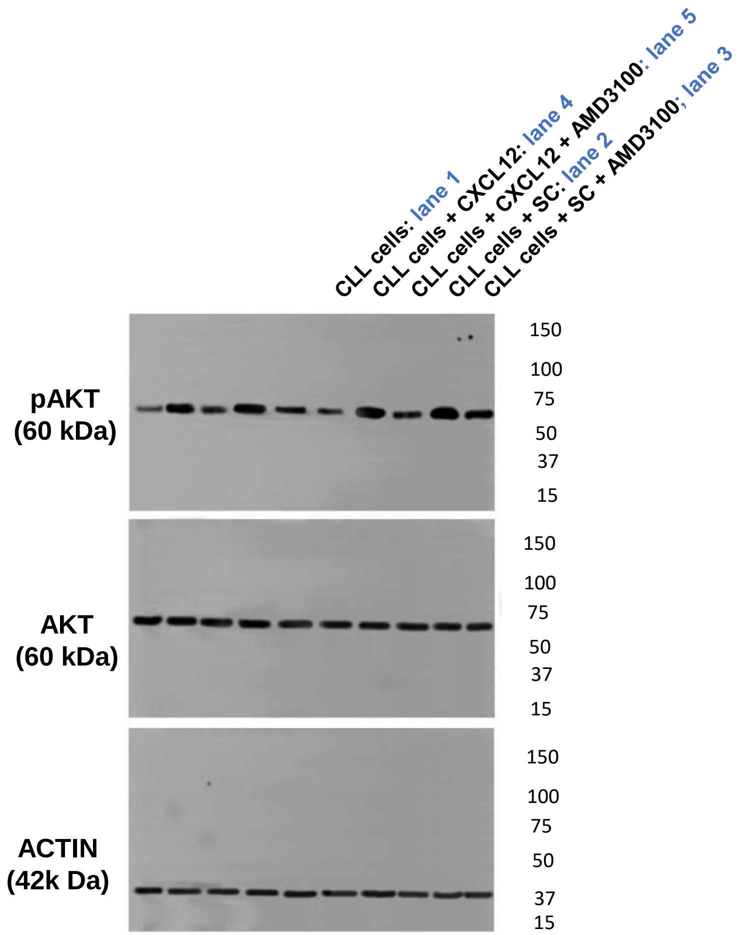

Densitometry reading

UPN15	-	SC	SC + AMD3100	CXCL12	CXCL12 + AMD3100
pAKT	99,47	157,11	129,70	148,78	102,78
ACTIN	99,12	96,77	93,36	114,46	89,67

The lanes of the whole blot used for each figure are indicated

Western blots

Figure 1, Panel D

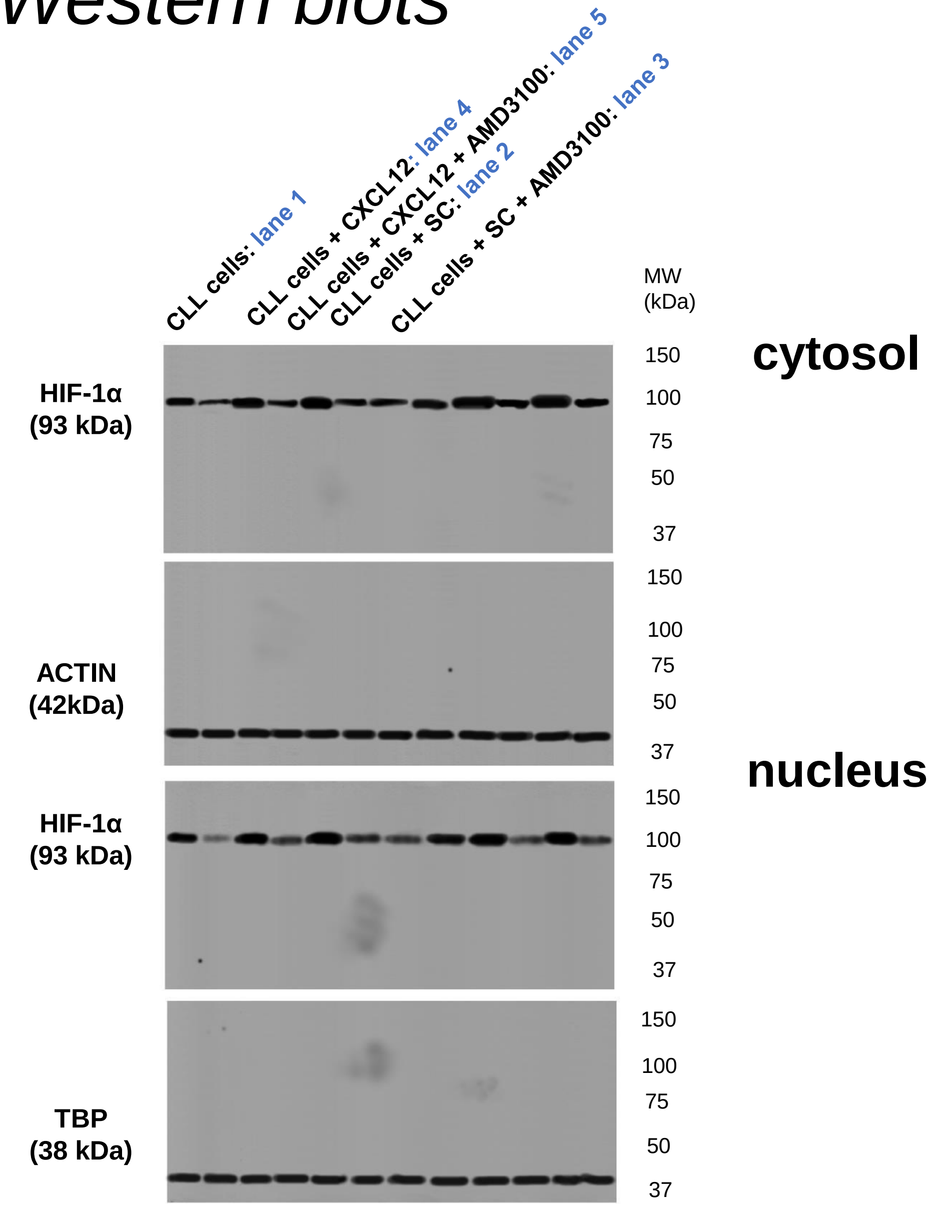

Densitometry reading

UPN15	-	SC	SC + AMD3100	CXCL12	CXCL12 + AMD3100
HIF-1a (C)	104,45	150,06	97,10	138,07	85,18
ACTIN	117,34	117,19	120,29	114,44	115,38
HIF-1a (N)	114,10	148,90	82,6	138,2	79
TBP	106,68	109,20	113,53	107,38	111,39

The lanes of the whole blot used for each figure are indicated

Western blots

Figure 2, Panels B, C

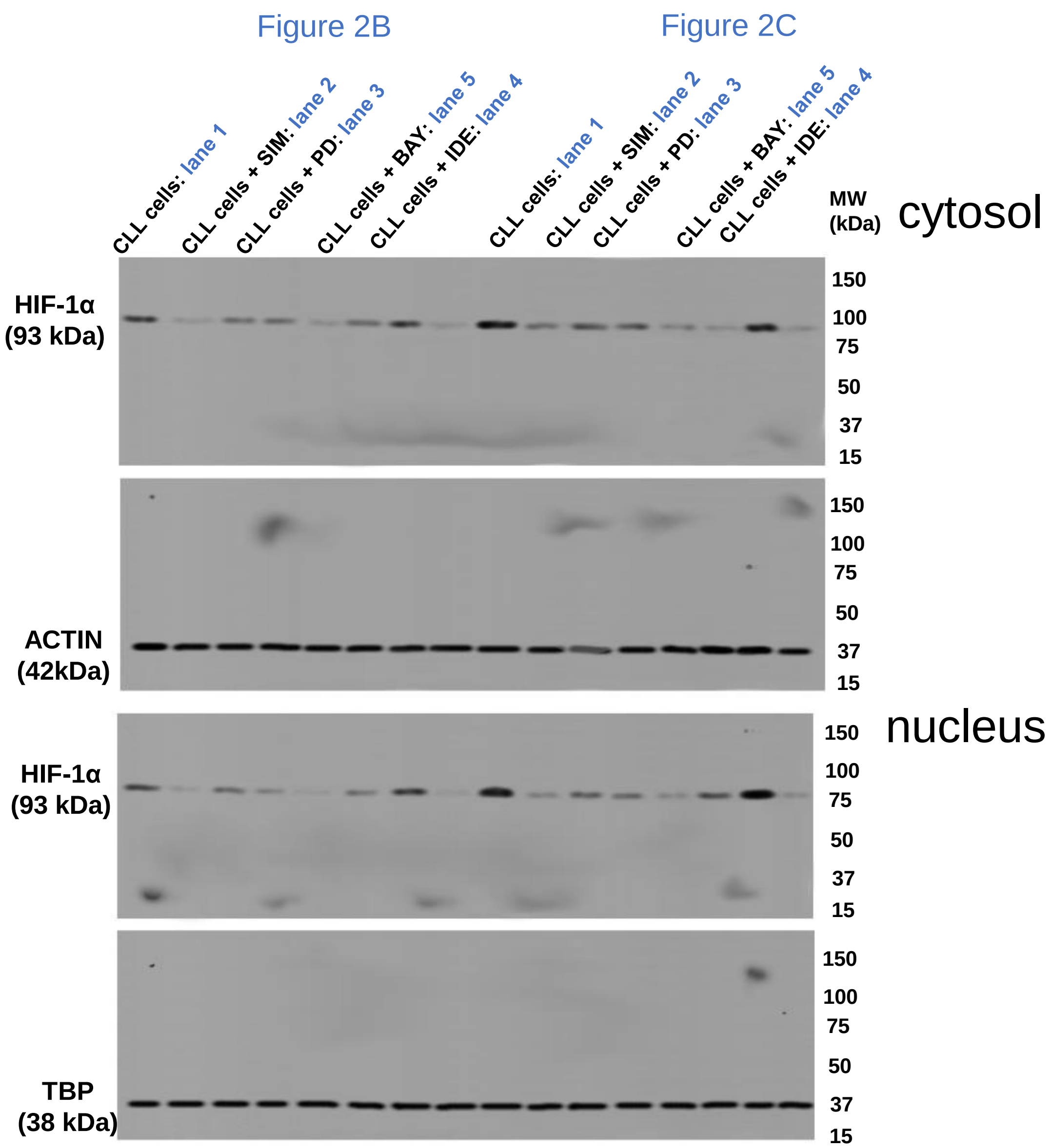

Densitometry reading

UPN79	-	SIM	PD	IDE	BAY
HIF-1a (C)	56,62	8,94	28,4	33	12,18
ACTIN	119,21	104,71	105,64	118,10	118,21
HIF-1a (N)	53,09	6,45	31,07	24,51	7,75
TBP	112,60	121,41	121,99	121,63	121,76

Densitometry reading

UPN79	SC	SC + SIM	SC + PD	SC + IDE	SC + BAY
HIF-1a (C)	101,97	32,52	53,40	16,56	23,41
ACTIN	120,06	113,54	113,79	134,07	120,58
HIF-1a (N)	100,21	20	38,71	47,78	17,66
TBP	122,68	119,41	126,80	120,39	120,78

The lanes of the whole blot used for each figure are indicated

Western blots

Figure 2, Panel D

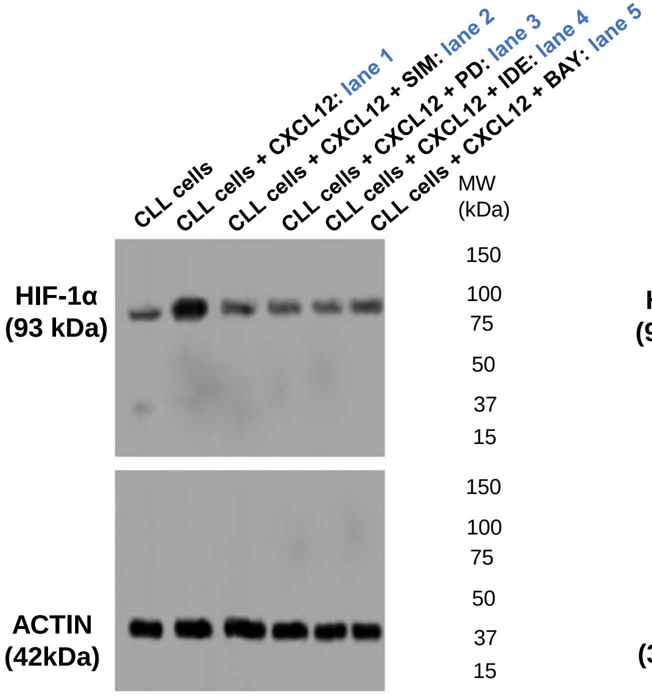

cytosol

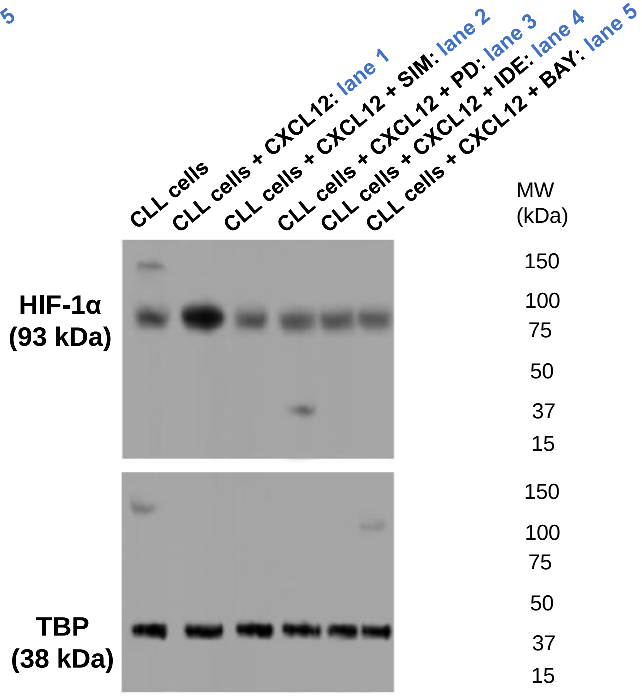

nucleus

Densitometry reading

UPN05	-	CXCL12	CXCL12 + SIM	CXCL12 + PD	CXCL12 + IDE	CXCL12 + BAY
HIF-1a (C)	52,30	129,31	65,05	55,24	44,88	62,60
ACTIN	133,83	131,88	141,20	137,47	136,22	134,88
HIF-1a (N)	64,01	131,86	56,88	59,48	57,34	52,27
TBP	133,10	129,13	131,73	118,32	118,34	129,63

The lanes of the whole blot used for each figure are indicated

Western blots

Figure 4, Panels A,B

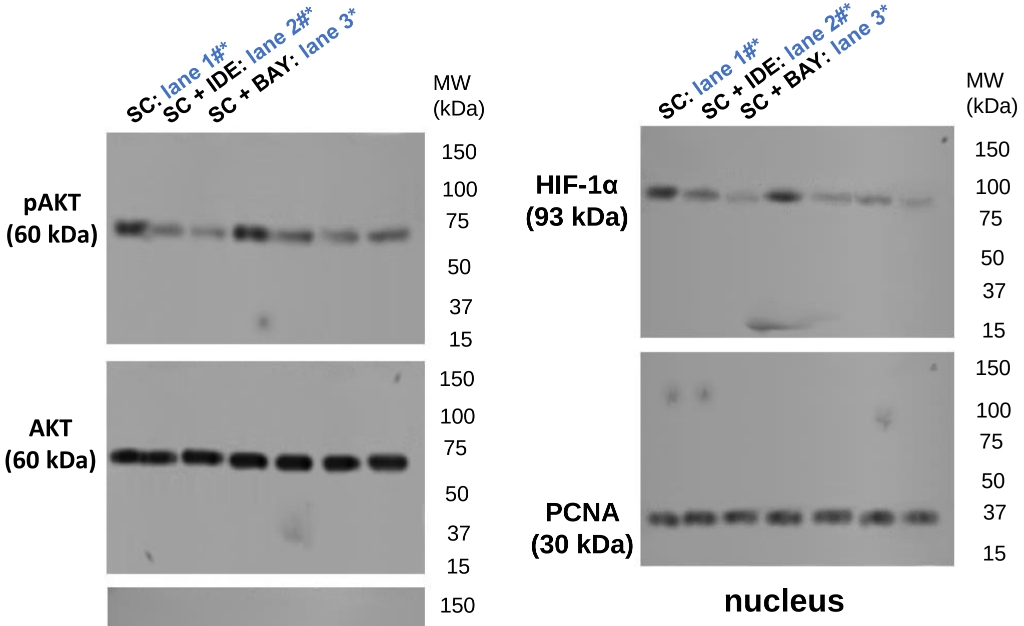

#: lanes present in Fig. 4A (pAkt, Akt, GAPDH)

*: lanes present in Fig. 4B (HIF-1α, GAPDH, PCNA)

Densitometry reading

M2-10B4	SC	SC + IDE	SC + BAY
pAKT	83,77	40,11	/
HIF-1a (C)	70,86	14,21	3,18
GAPDH	105,01	122,38	135,24
HIF-1a (N)	59,63	31,85	11,03
PCNA	64,59	73,68	69,06

The lanes of the whole blot used for each figure are indicated

Western blots

Figure 4, Panels A,B

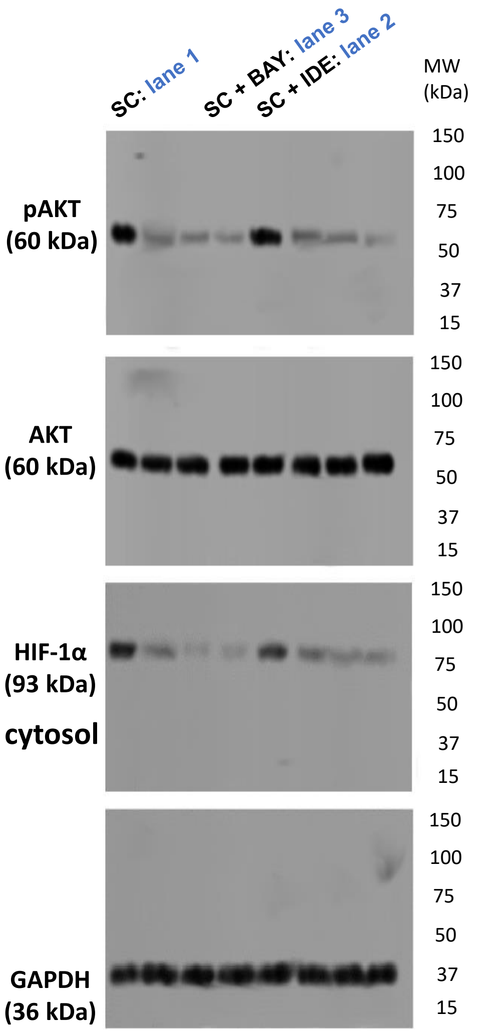

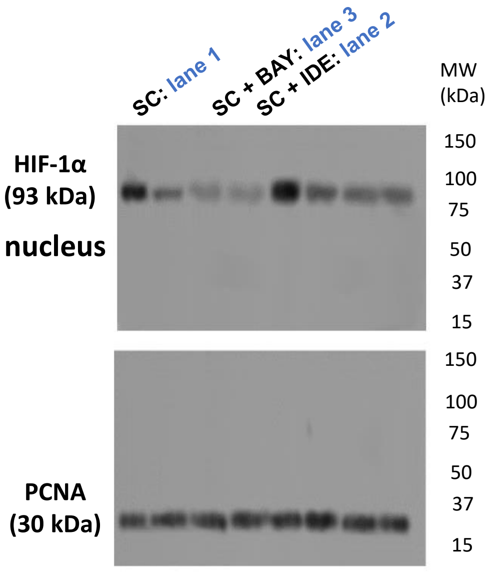

#: lanes present in Fig. 4A (pAkt, Akt, GAPDH)

*: lanes present in Fig. 4B (HIF-1α, GAPDH, PCNA)

Densitometry reading

UPN31	SC	SC + IDE	SC + BAY
pAKT	110,38	18,90	/
HIF-1a (C)	87,78	10,07	9,41
GAPDH	97,86	112,54	115,66
HIF-1a (N)	91,16	20,96	25,65
PCNA	54,71	69,74	68,85

The lanes of the whole blot used for each figure are indicated

Western blots

Figure 6, Panel A

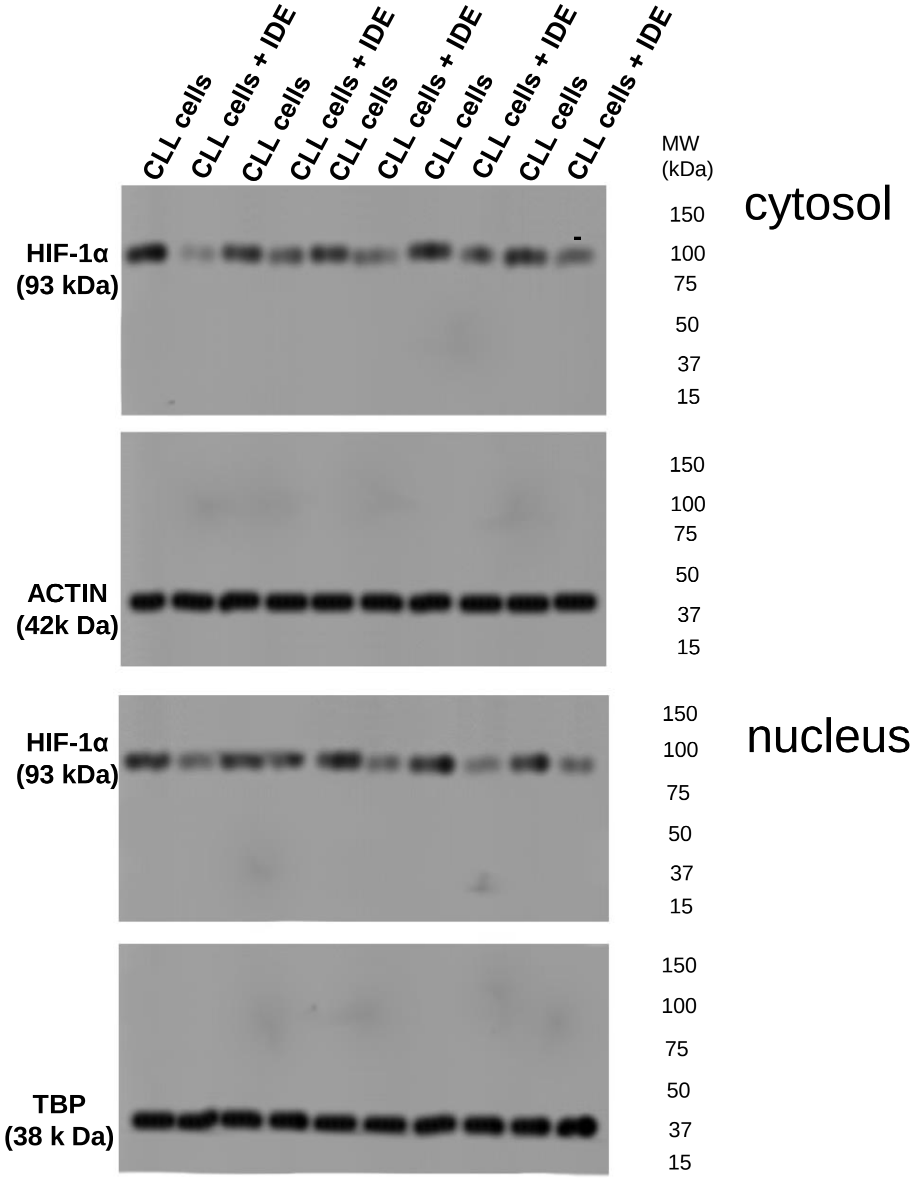

All lanes of the blot are included in the figure

Densitometry reading

UPN86	-	IDE
HIF-1a (C)	82,62	18,36
ACTIN	92,79	105,72
HIF-1a (N)	73,55	37,64
TBP	107,18	106,82

UPN76	-	IDE
HIF-1a (C)	68,11	41,12
ACTIN	106,05	106,70
HIF-1a (N)	81,74	37,20
TBP	106,19	106,91

UPN11	-	IDE
HIF-1a (C)	73,70	43,24
ACTIN	107	106,54
HIF-1a (N)	70,55	36,50
TBP	105,47	116,48

Densitometry reading

UPN06	-	IDE
HIF-1a (C)	64,04	47,09
ACTIN	106,01	106,58
HIF-1a (N)	68,64	58,98
TBP	107,01	105,34

UPN70	-	IDE
HIF-1a (C)	79,27	48,31
ACTIN	106,46	106,60
HIF-1a (N)	78,77	29,72
TBP	105,92	106,01

Western blots

Figure S1

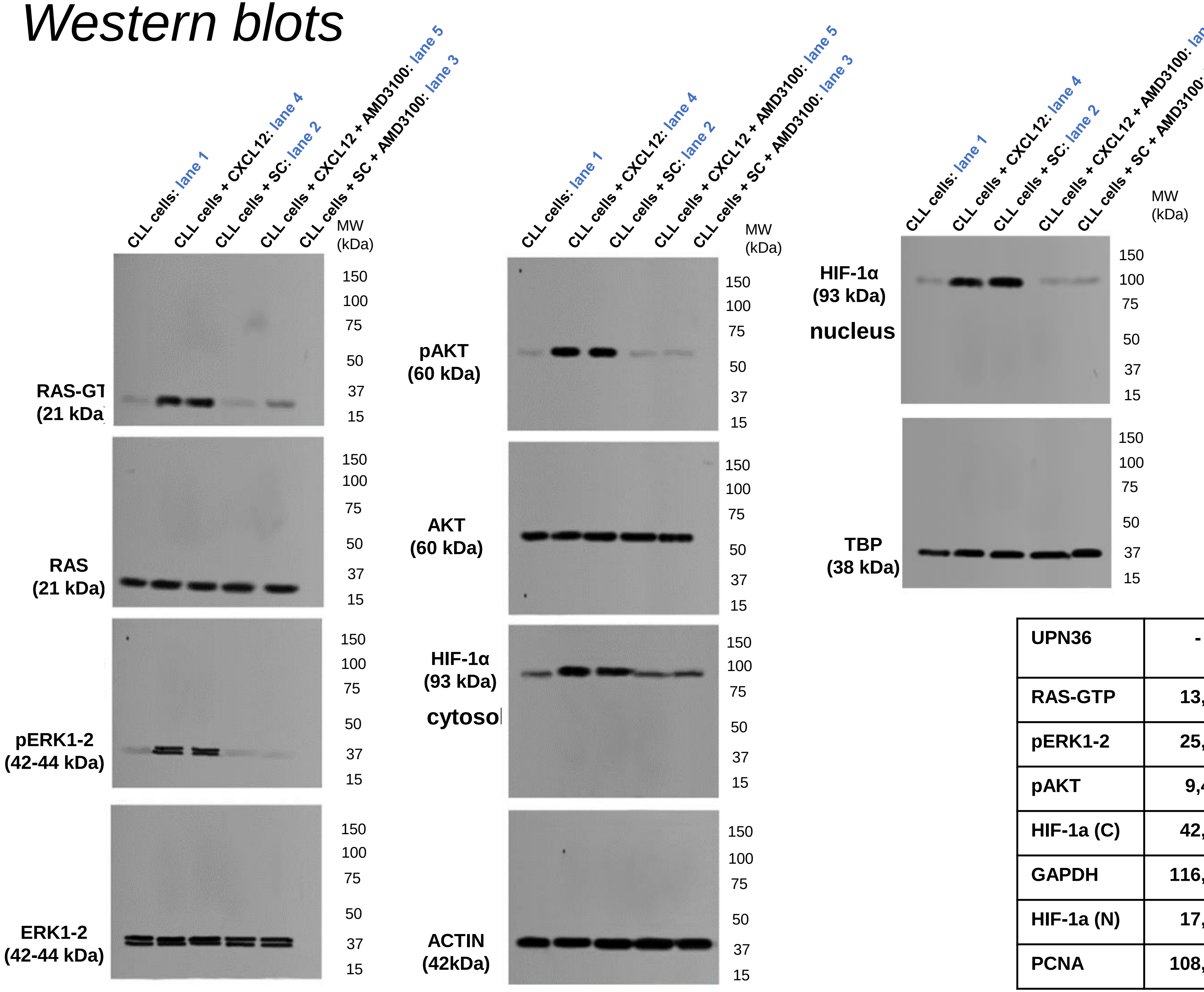

The lanes of the whole blot used for each figure are indicated

Densitometry reading

UPN36	-	SC	SC + AMD3100	CXCL12	CXCL12 + AMD3100
RAS-GTP	13,7	82,5	17,2	77,5	2,9
pERK1-2	25,7	84,4	7,4	91,9	13,8
pAKT	9,4	101,2	1,9	104,7	2,9
HIF-1a (C)	42,7	96,5	50,2	112,4	47,7
GAPDH	116,60	127,06	127,11	123,36	128,09
HIF-1a (N)	17,1	122,1	10,4	103,9	10,4
PCNA	108,14	141,27	133,92	133,04	126,53

Western blots

Figure S3, Panels A,B

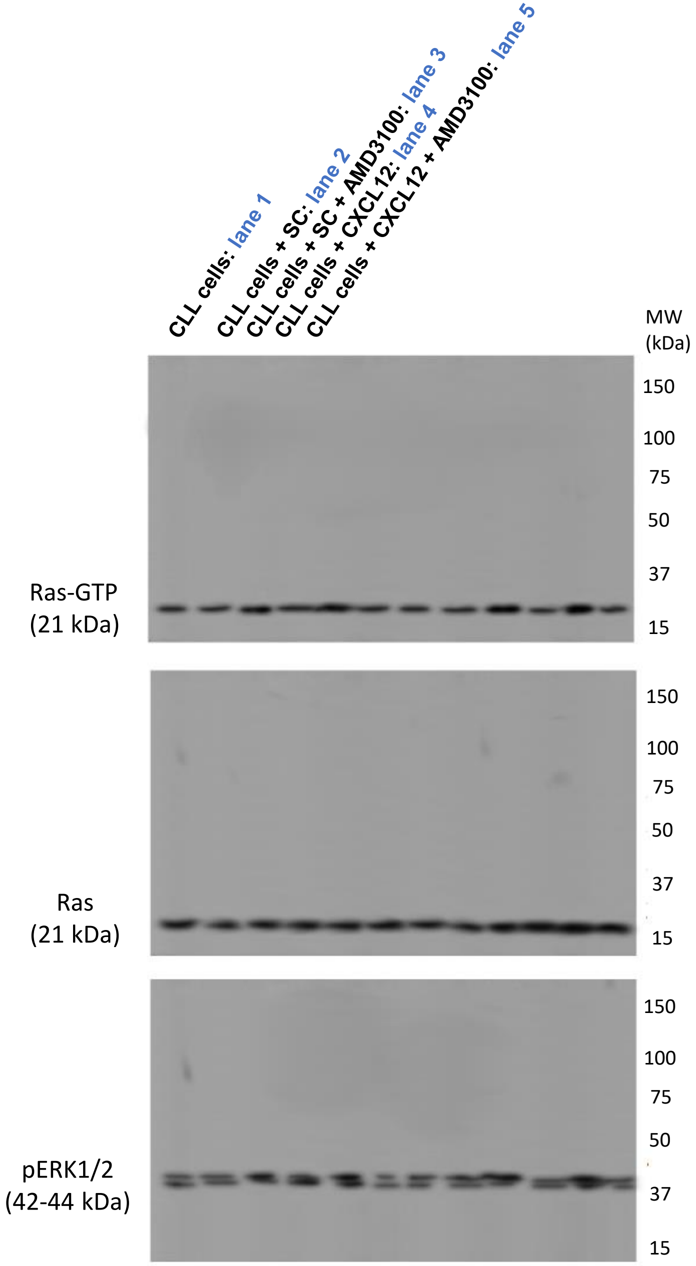

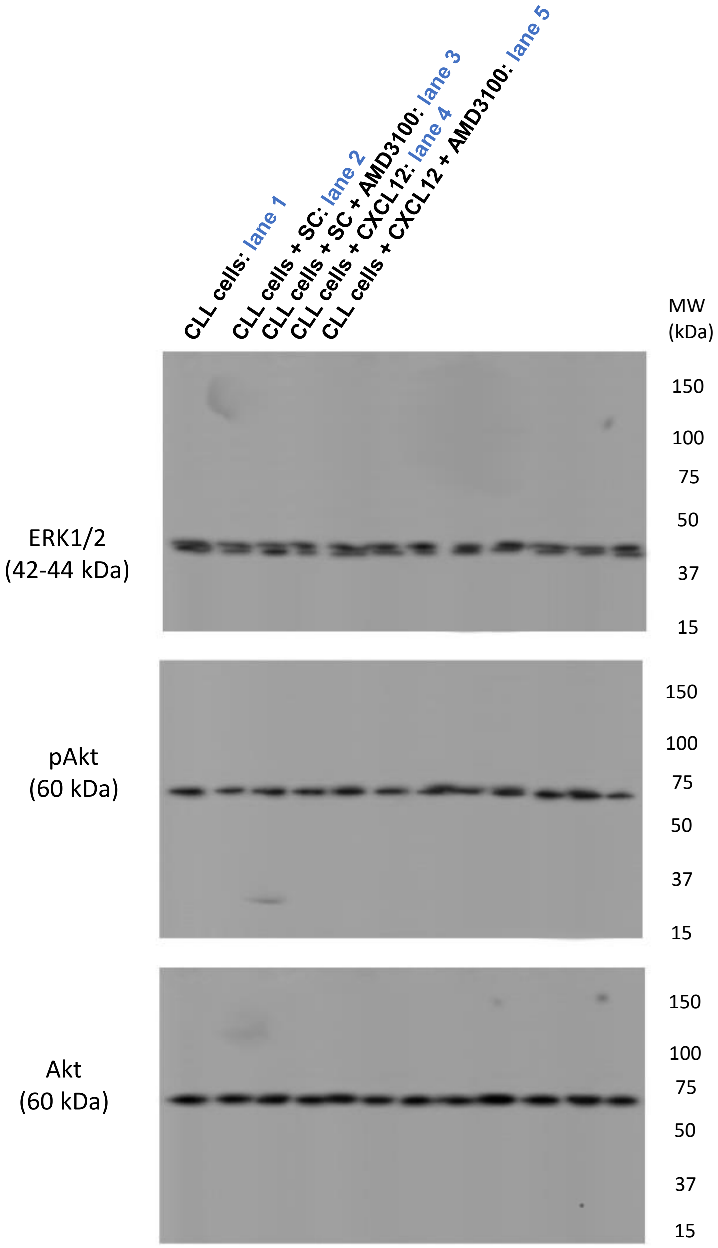

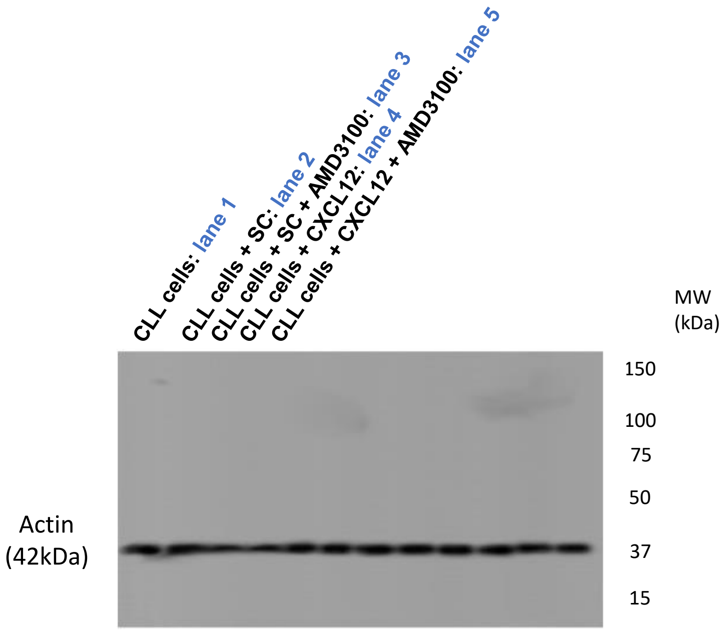

Densitometry reading

UPN08	-	SC	SC + AMD3100	CXCL12	CXCL12 + AMD3100
RAS-GTP	74,54	101,83	86,84	109,86	80,97
pERK1-2	56,09	75,60	83,37	95,80	52,97
pAKT	86,32	87,07	84,79	97,98	79,44
ACTIN	111,48	111,51	106,93	119,30	119,01

The lanes of the whole blot used for each figure are indicated

Western blots

Figure S3, Panel D

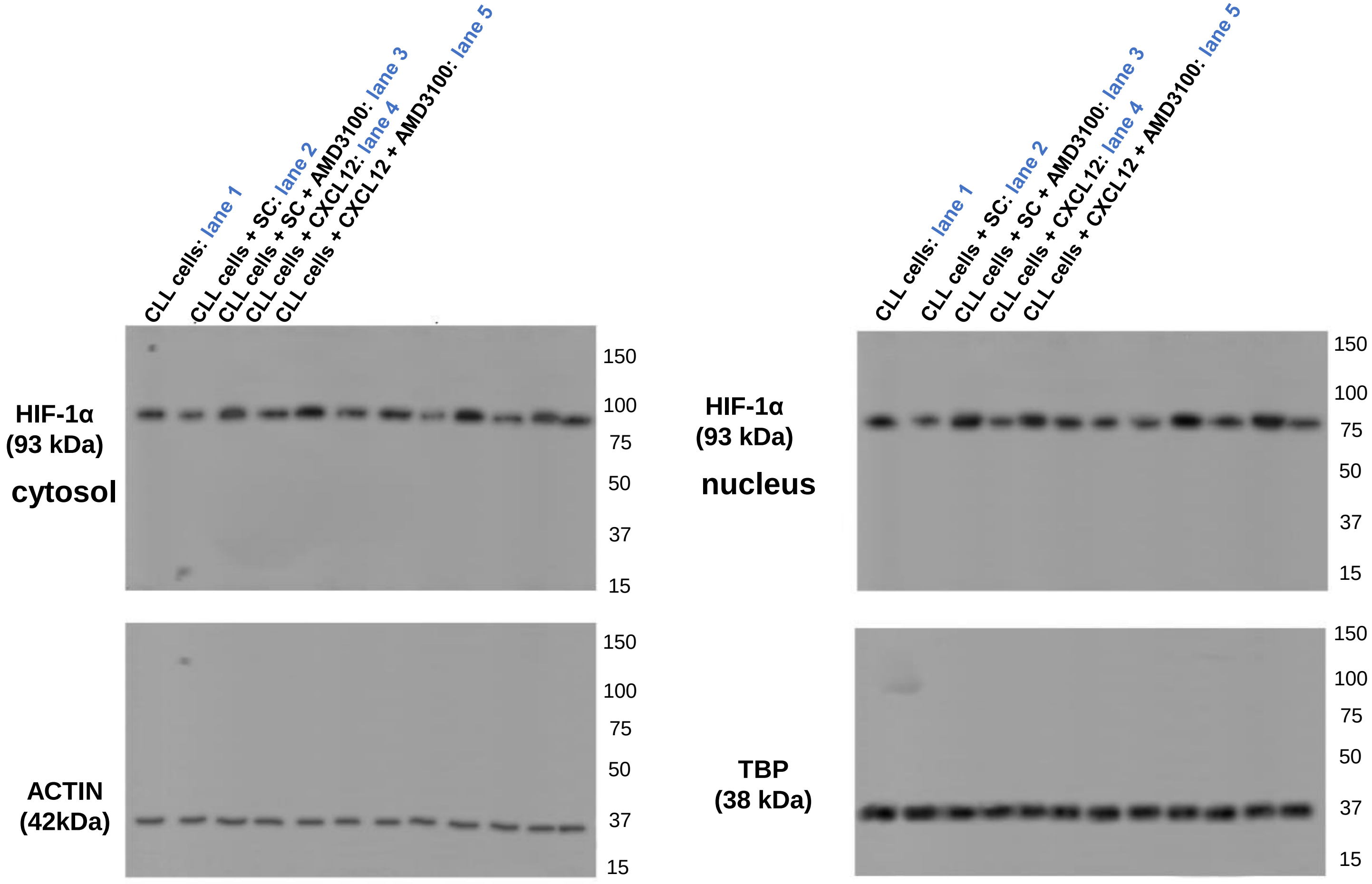

Densitometry reading

UPN08	-	SC	SC + AMD3100	CXCL12	CXCL12 + AMD3100
HIF-1a (C)	63,96	71,01	65,21	92,30	59,38
ACTIN	58,44	60,75	59,58	57,93	63,12
HIF-1a (N)	90,56	106,38	61,12	96,02	83,61
TBP	103,35	112,17	104,32	101,43	104,78

The lanes of the whole blot used for each figure are indicated

Western blots

Figure S4

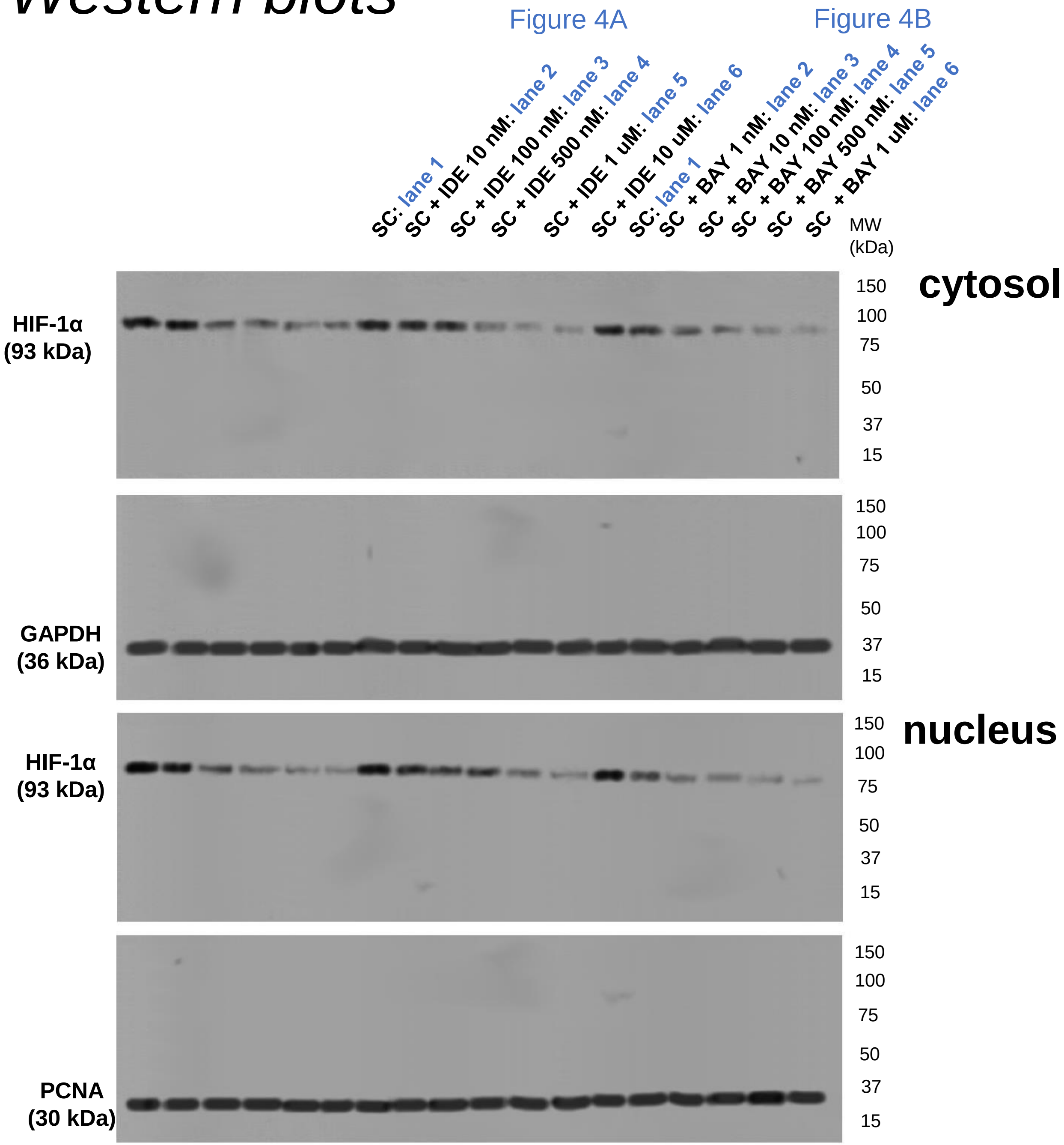

Densitometry reading

M2-10B4	SC	SC + IDE 10 nM	SC + IDE 100 nM	SC + IDE 500 nM	SC + IDE 1 uM	SC + IDE 10 uM
HIF-1a (C)	87,04	73,26	72,12	31,07	15,71	16,91
GAPDH	91,37	92,03	89,72	92,77	87,97	89,53
HIF-1a (N)	106,85	81,09	66,13	68,69	37,45	20,96
PCNA	106,7	103,69	108,24	102,92	102,72	107,62

M2-10B4	SC	SC + BAY 1 nM	SC + BAY 10 nM	SC + BAY 100 nM	SC + BAY 500 nM	SC + BAY 1 uM
HIF-1a (C)	80,11	70,24	44,08	30,06	18,90	13,42
GAPDH	87,41	90,56	88,39	91,06	92,20	92,23
HIF-1a (N)	99,57	56,86	31,81	28,42	18,59	12,54
PCNA	100,37	105,32	99,84	102,89	118,84	96,29
