# Supplementary material for: Targeting HIF-1α Regulatory Pathways as a Strategy to Hamper Tumor-Microenvironment Interactions in CLL
Source: Cancers (Basel). 2021 Jun 9;13(12):2883. doi: 10.3390/cancers13122883 (PMC8229189; doi:10.3390/cancers13122883)
Supplement: Supplementary file 1 [file cancers-13-02883-s001.zip › cancers-1223225pd-supplementary.pdf]

Supplementary Information

Targeting HIF-1α Regulatory Pathways as a Strategy to Hamper Tumor-Microenvironment Interactions in CLL

**Candida Vitale [1,2,†], Valentina Griggio [1,2,†], Chiara Riganti [3], Maria Todaro [1,2], Joanna Kopecka [3], Rebecca Jones [1,2], Chiara Salvetti [1,2], Elia Boccellato [1,2], Francesca Perutelli [1,2], Claudia Voena [2], Laura Godio [4], Mario Boccadoro [1,2]
and Marta Coscia [1,2,*]**

[1] University Division of Hematology, A.O.U. Città della Salute e della Scienza di Torino, via Genova 3,
10126 Torino, Italy; candida.vitale@unito.it (C.V.); valentina.griggio@unito.it (V.G.); maria.todaro@unito.it (M.T.); rebecca.jones@unito.it (R.J.); chiara.salvetti@unito.it (C.S.); elia.boccellato@unito.it (E.B.); francesca.perutelli@unito.it (F.P.); mario.boccadoro@unito.it (M.B.)

[2] Department of Molecular Biotechnology and Health Sciences, University of Torino, via Nizza 52,
10126 Torino, Italy; claudia.voena@unito.it

[3] Department of Oncology, University of Torino, via Santena 5, 10126 Torino, Italy; chiara.riganti@unito.it (C.R.); joanna.kopecka@unito.it (J.K.)

[4] Division of Pathology, A.O.U. Città della Salute e della Scienza di Torino, via Santena 5, 10126 Torino, Italy; lgodio@cittadellasalute.to.it

* Correspondence: marta.coscia@unito.it; Tel.: +39-0116336728; Fax: +39-0116963737

† These authors contributed equally to this work.

Table S1. Summary of patients' characteristics

Unique Patient Number (UPN)	Sex	IGHV	Del(17p) and/or *TP53* mutation	Therapy
UPN01	m	M	N	off-therapy
UPN02	f	M	Y	off-therapy
UPN03	m	UM	Y	off-therapy
UPN04	f	UM	Y	off-therapy
UPN05	m	UM	N	off-therapy
UPN07	f	M/UM	N	off-therapy
UPN08	f	M	N*	off-therapy
UPN09	m	UM	N	off-therapy
UPN10	f	UM	Y	off-therapy
UPN12	m	M	N	off-therapy
UPN13	m	M	N*	off-therapy
UPN14	m	UM	Y	off-therapy
UPN15	m	UM	N	off-therapy
UPN16	m	UM	N	off-therapy
UPN17	m	UM	N	off-therapy
UPN18	m	M	Y	off-therapy
UPN19	m	M	N	off-therapy
UPN20	m	M	N*	off-therapy
UPN21	m	UM	N	off-therapy
UPN22	f	na	Y	off-therapy
UPN23	m	UM	N	off-therapy
UPN24	m	na	N	off-therapy
UPN25	m	UM	N	off-therapy
UPN26	m	M	Y	off-therapy
UPN27	m	UM	N*	off-therapy
UPN28	f	M	Y	off-therapy
UPN29	m	UM	N	off-therapy
UPN30	m	UM	N	off-therapy
UPN31	m	UM	N	off-therapy
UPN32	m	UM	Y	off-therapy
UPN33	f	na	na	off-therapy
UPN34	m	M	N	off-therapy
UPN35	m	na	Y	off-therapy
UPN36	m	UM	N	off-therapy
UPN37	m	UM	N	off-therapy
UPN38	m	UM	N	off-therapy
UPN39	f	M	N	off-therapy
UPN40	m	M	N	off-therapy
UPN41	m	UM	N	off-therapy
UPN42	f	UM	N	off-therapy
UPN43	f	na	Y	off-therapy
UPN44	m	M	N*	off-therapy

UPN45	m	UM	N	off-therapy
UPN46	m	UM	Y	off-therapy
UPN47	m	na	na	off-therapy
UPN48	f	M	N	off-therapy
UPN49	m	M	N	off-therapy
UPN50	m	UM	N	off-therapy
UPN51	f	UM	Y	off-therapy
UPN52	m	UM	Y	off-therapy
UPN53	f	UM	N	off-therapy
UPN54	f	UM	N	off-therapy
UPN55	f	na	Y	off-therapy
UPN56	m	M	Y	off-therapy
UPN57	f	M	Y	off-therapy
UPN58	m	na	Y	off-therapy
UPN59	m	UM	N	off-therapy
UPN60	f	M	N	off-therapy
UPN61	f	UM	N	off-therapy
UPN62	m	M	N	off-therapy
UPN63	m	na	na	off-therapy
UPN64	m	na	N	off-therapy
UPN65	f	UM	Y	off-therapy
UPN66	m	M	N	off-therapy
UPN67	f	M	N	off-therapy
UPN68	m	M	Y	off-therapy
UPN69	m	UM	N	off-therapy
UPN71	m	UM	N	off-therapy
UPN72	m	UM	Y	off-therapy
UPN73	f	na	na	off-therapy
UPN74	m	na	Y	off-therapy
UPN75	m	UM	Y	off-therapy
UPN77	m	na	N	off-therapy
UPN78	f	UM	N	off-therapy
UPN79	m	M	N	off-therapy
UPN80	f	UM	N	off-therapy
UPN81	m	na	na	off-therapy
UPN82	m	UM	N*	off-therapy
UPN83	m	UM	N	off-therapy
UPN84	m	M	N	off-therapy
UPN85	f	UM	N	off-therapy
UPN87	f	na	N*	off-therapy
UPN88	f	UM	N	off-therapy
UPN89	m	na	N	off-therapy
UPN90	m	M	N	off-therapy
UPN91	m	na	na	off-therapy
UPN92	m	UM	N	off-therapy
UPN93	m	na	na	off-therapy
UPN94	m	M	N	off-therapy

UPN95	m	na	N*	off-therapy
UPN96	f	UM	N	off-therapy
UPN97	m	UM	Y	off-therapy
UPN98	m	na	na	off-therapy
UPN99	m	M	N	off-therapy
UPN100	m	M	N*	off-therapy
UPN101	f	M	N	off-therapy
UPN102	m	UM	N	off-therapy
UPN103	m	na	N*	off-therapy
UPN104	f	M	N*	off-therapy
UPN105	f	UM	N	off-therapy
UPN06	m	UM	N	idelalisib
UPN11	f	M	N	idelalisib
UPN70	f	M	N	idelalisib
UPN76	f	UM	Y	idelalisib
UPN86	m	UM	Y	idelalisib

Abbreviations: UPN, unique patient number; m, male; f, female; IGHV, immunoglobulin heavy chain variable region; M, mutated; UM, unmutated; na, not available; * Del(17p) is not present as assessed by fluorescence in situ hybridization, data on *TP53* mutation is not available.

Figure S1

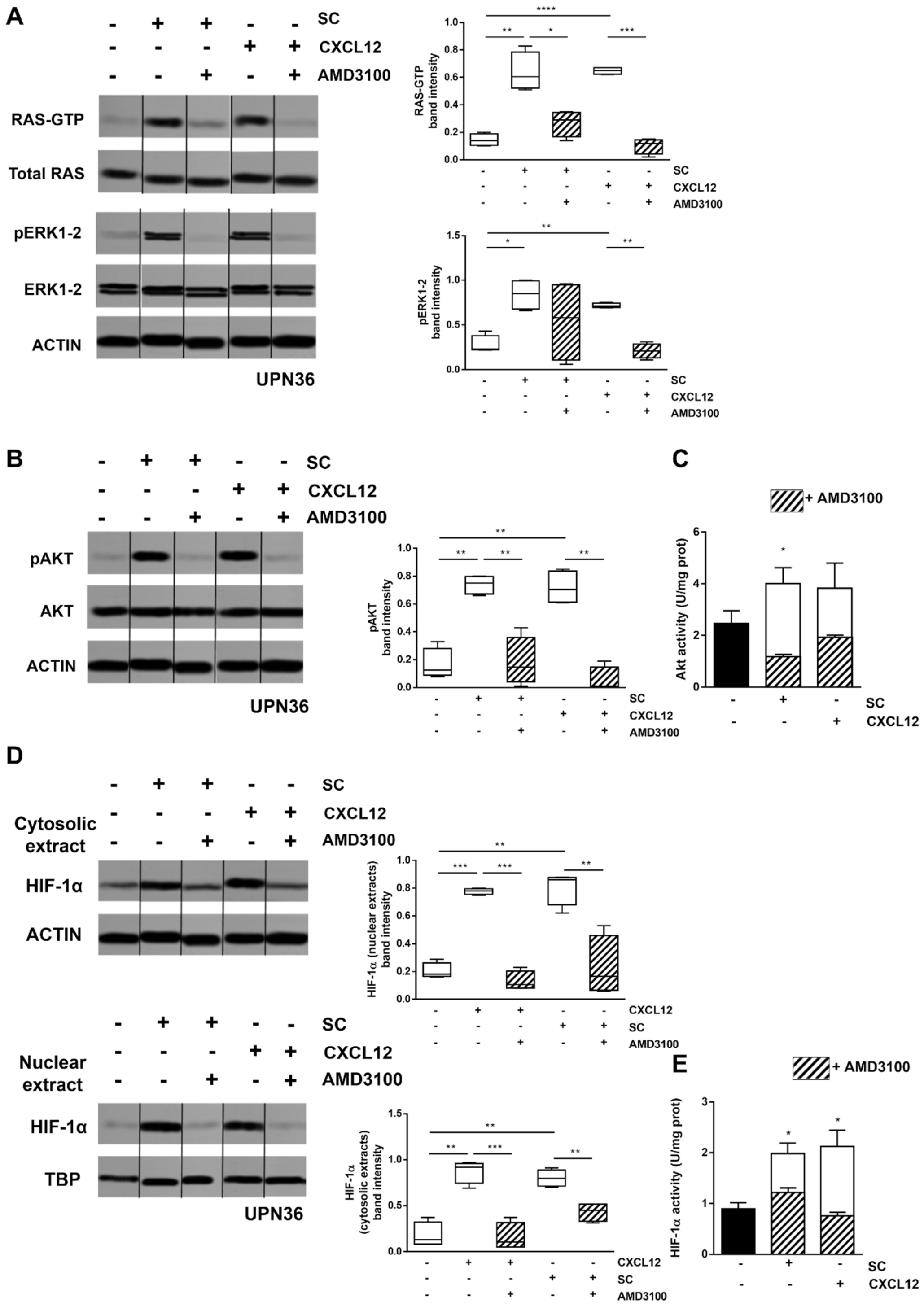
A
SC
CXCL12
AMD3100
RAS-GTP
Total RAS
pERK1-2
ERK1-2
ACTIN
UPN36
RAS-GTP band intensity
pERK1-2 band intensity
B
pAKT
AKT
pAKT band intensity
C
+ AMD3100
Akt activity (U/mg prot)
D
Cytosolic extract
HIF-1α
Nuclear extract
TBP
HIF-1α (nuclear extracts) band intensity
HIF-1α (cytosolic extracts) band intensity
E
HIF-1α activity (U/mg prot)

Figure S1. The CXCL12/CXCR4 axis plays a central role in the SC-mediated triggering of HIF-1α regulatory pathways. Primary CLL cells were cultured for 6 hours in presence of M2-10B4 SC or CXCL12. In selected conditions, the CXCR4 antagonist AMD3100 was added. Both SC and CXCL12 induced an increase in the amount of GTP-bound RAS (RAS-GTP) and of the active phosphorylated form of ERK1-2 (pERK1-2) (**A**), and in the phosphorylation and activity of AKT (**B**,**C**). Accordingly, CLL cells cultured with SC or CXCL12 displayed an increase in the cytosolic and nuclear amount of HIF-1α (**D**), and in HIF-1α activity (**E**). The addition of the CXCR4 antagonist AMD3100 abrogated the inducing effects mediated both by SC and CXCL12 at all levels, except for pERK1-2 when CLL cells were exposed to SC. In (**A**,**B**,**D**) a representative blot (with relative Unique Patient Number, UPN) together with the corresponding cumulative band intensity data of 4 independent experiments is shown. Box and whiskers plots represent median values, 25%-75% percentiles, and minimum and maximum values for each group. In (**C**,**E**) bar graphs represent mean results and SEM (n = 4). Vertical lines have been inserted to indicate repositioned gel lanes. **** $p<0.0001$, *** $p<0.001$, ** $p<0.01$ and *$p<0.05$. Please find the whole western blot in the supplementary file 1.

Figure S2

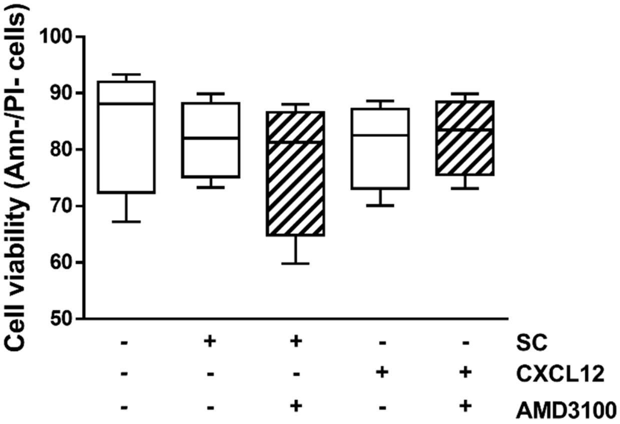

Figure S2. The exposure of CLL cells to AMD3100 does not reduce tumor cell viability. Primary CLL cells were cultured for 6 hours in presence of M2-10B4 SC or CXCL12. In selected conditions, the CXCR4 antagonist AMD3100 was added. There is no significant difference in the viability of CLL cells cultured in different conditions. Box and whiskers plots represent median values, 25%-75% percentiles, and minimum and maximum values for each group (n = 4).

Figure S3

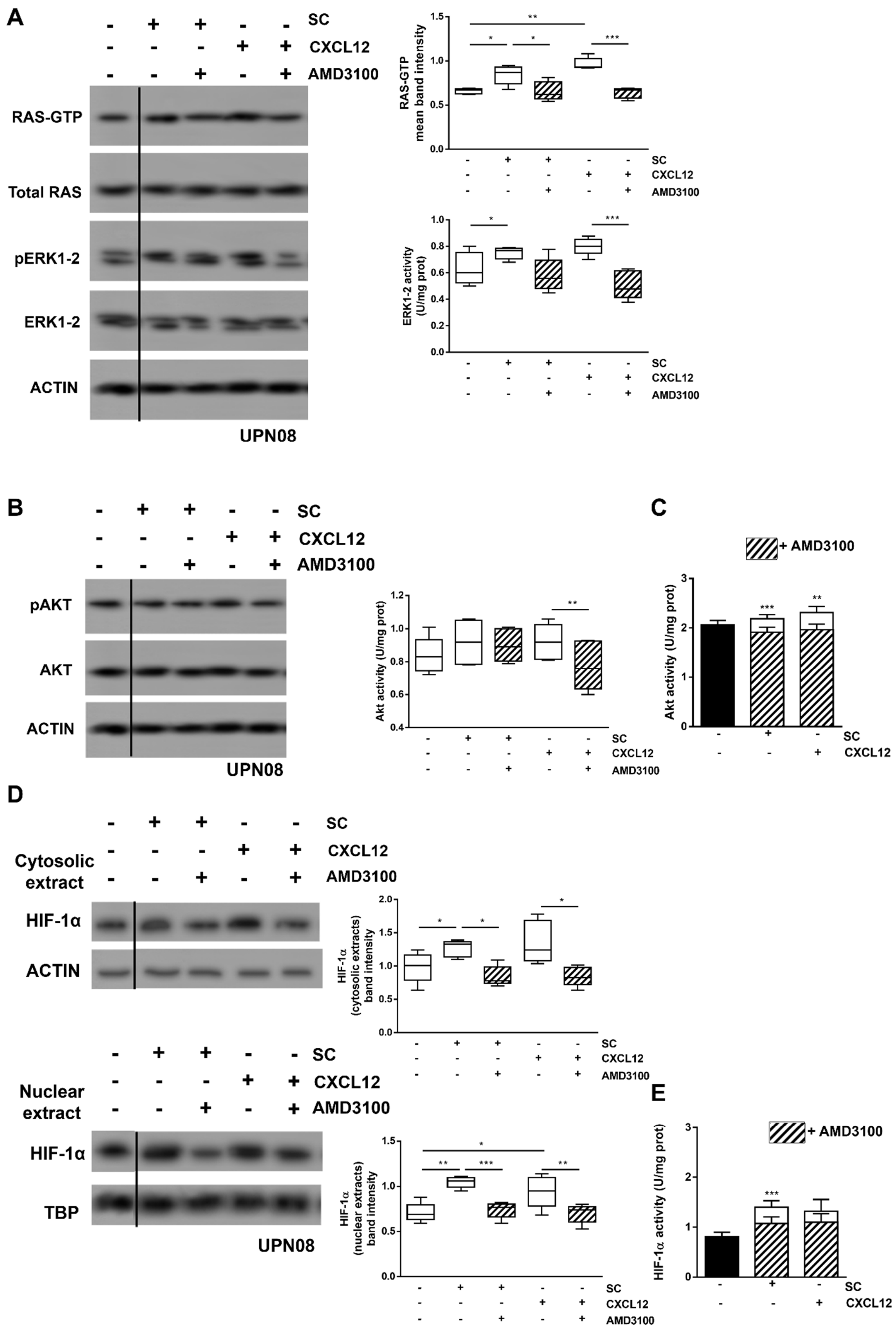
A
SC
CXCL12
AMD3100
RAS-GTP
Total RAS
pERK1-2
ERK1-2
ACTIN
UPN08
RAS-GTP mean band intensity
ERK1-2 activity (U/mg prot)
B
pAKT
AKT
ACTIN
UPN08
Akt activity (U/mg prot)
C
+ AMD3100
Akt activity (U/mg prot)
D
Cytosolic extract
HIF-1α
ACTIN
HIF-1α (cytosolic extracts) band intensity
Nuclear extract
HIF-1α
TBP
UPN08
HIF-1α (nuclear extracts) band intensity
E
+ AMD3100
HIF-1α activity (U/mg prot)

Figure S3. The CXCL12/CXCR4 axis plays a central role in the SC-mediated triggering of HIF-1α regulatory pathways. Primary CLL cells were cultured for 30 minutes in presence of M2-10B4 SC or CXCL12. In selected conditions, the CXCR4 antagonist AMD3100 was added. Both SC and CXCL12 induced an increase in the amount of GTP-bound RAS (RAS-GTP) and of the active phosphorylated form of ERK1-2 (pERK1-2) (**A**). By contrast, the phosphorylation and activity of AKT was not significantly modulated by the microenvironmental stimuli, at this early timepoint (**B-C**). CLL cells cultured with SC or CXCL12 displayed an increase in the cytosolic and nuclear amount of HIF-1α (**D**), and in HIF-1α activity (**E**). The addition of the CXCR4 antagonist AMD3100 abrogated the upregulation induced by SC and CXCL12 on RAS, ERK1-2 and HIF-1α. In (**A,B,D**) a representative blot (with relative Unique Patient Number, UPN) together with the corresponding cumulative band intensity data of 5 independent experiments is shown. Box and whiskers plots represent median values, 25%-75% percentiles, and minimum and maximum values for each group. In (**C,E**) bar graphs represent mean results and SEM (n = 5). Vertical lines have been inserted to indicate repositioned gel lanes. **** $p<0.0001$, *** $p<0.001$, ** $p<0.01$ and *$p<0.05$. Please find the whole western blot in the supplementary file 1.

Figure S4

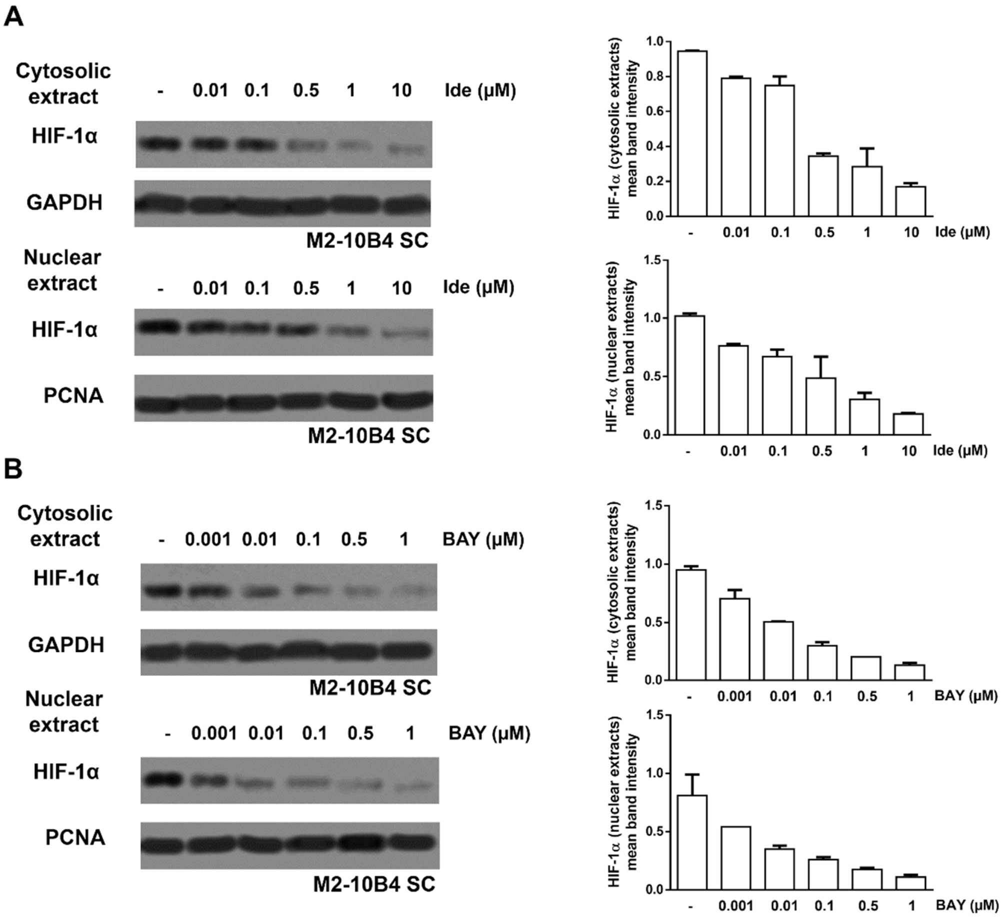

Figure S4. Increasing concentrations of idelalisib or BAY87-2243 determine a progressive reduction of HIF-1α levels in SC. M2-10B4 SC were cultured for 48 hours in the presence of increasing concentration of idelalisib (Ide, 0.01 µM, 0.1 µM, 0.5 µM, 1 µM or 10 µM) (**A**), or BAY87-2243 (BAY, 0.001 µM, 0.01 µM, 0.1 µM, 0.5 µM or 1 µM) (**B**). WB analyses of HIF-1α protein expression for 1 representative experiment out of 2 and the corresponding cumulative band intensity data are shown. Please find the whole western blot in the supplementary file 1.
